# Synergistic Disruption of Survival and Metastatic Potential in Esophageal Adenocarcinoma Cells Through Combined Inhibition of HIF1α and CD73

**DOI:** 10.3390/cancers17244016

**Published:** 2025-12-17

**Authors:** Ian Mersich, Alexander Malmberg, Eahsanul Anik, Md Sazzad Hassan, Urs von Holzen, Brian S. J. Blagg, Aktar Ali

**Affiliations:** 1 Department of Chemistry and Biochemistry, University of Notre Dame, Notre Dame, IN 46556, USA; imersich@nd.edu (I.M.);; 2 Warren Family Research Center for Drug Discovery and Development, University of Notre Dame, Notre Dame, IN 46556, USA; 3 Mike and Josie Harper Cancer Research Institute, South Bend, IN 46617, USA; 4 Department of Surgery, Indiana University School of Medicine, South Bend, IN 46617, USA; 5 Goshen Center for Cancer Care, Goshen, IN 46526, USA

**Keywords:** HIF1α, CD73, NT5E, hypoxia, esophageal adenocarcinoma (EAC), purinergic signaling, angiogenesis

## Abstract

Background/Objectives: Hypoxia promotes esophageal adenocarcinoma (EAC) aggressiveness through stabilization of hypoxia-inducible factor-1α (HIF-1α), which regulates pro-survival, pro-metastatic, and immunosuppressive pathways, including the ectoenzyme CD73 (NT5E). Although CD73 is a known hypoxia-responsive gene, its functional integration with HIF-1α signaling in EAC remains incompletely understood. This study aimed to define the relationship between HIF-1α and CD73 in EAC and to evaluate the therapeutic potential of their combined inhibition. Methods: Gene expression and survival analyses were performed using CCLE and TCGA-ESCA datasets. CD73 and HIF-1α expression were evaluated in EAC patient tissues by immunohistochemistry. EAC cell lines were subjected to hypoxic conditions with genetic or pharmacologic inhibition of HIF-1α and/or CD73. Cell viability, migration, angiogenesis, VEGF secretion, and purinergic metabolite levels were assessed using luminescence assays, Boyden chamber migration assays, endothelial tube formation assays, ELISA, and targeted LC-MS/MS, respectively. Results: NT5E expression was transcriptionally upregulated by HIF-1α under hypoxia and correlated with advanced disease stage and poor overall survival in EAC patients. While CD73 inhibition alone modestly reduced EAC cell viability, combined inhibition of HIF-1α and CD73 synergistically decreased tumor cell survival, particularly under hypoxic conditions, and significantly altered extracellular adenosine metabolism. Dual targeting further suppressed migration, reduced VEGF secretion, and impaired angiogenic signaling, indicating disruption of tumor microenvironmental pathways critical for metastasis and immune evasion. Conclusions: These findings identify CD73 as a direct hypoxia-responsive effector of HIF-1α in EAC and demonstrate that dual inhibition of HIF-1α and CD73 synergistically disrupts tumor cell survival and pro-metastatic signaling. This combinatorial strategy represents a mechanistically integrated therapeutic approach to overcome hypoxia-driven resistance in esophageal adenocarcinoma.

## Simple Summary

Hypoxia-driven stabilization of HIF1α promotes therapeutic resistance and metastasis in esophageal adenocarcinoma (EAC). This study identifies CD73 (NT5E) as a direct hypoxia-responsive effector of HIF1α and demonstrates that dual inhibition of HIF1α and CD73 synergistically suppresses tumor cell viability, disrupts adenosine metabolism, and impairs angiogenic and migratory signaling. These findings highlight a mechanistically integrated therapeutic strategy to target hypoxia-adapted, treatment-resistant EAC.

## 1. Introduction

In 2025 in the United States, ~22,070 new esophageal cancer cases and ~16,250 deaths are estimated [1], and worldwide in 2020, there were ~510,000 new cases of esophageal cancer and ~540,000 deaths globally [2], with esophageal adenocarcinoma (EAC) representing a rapidly rising subtype in Western countries due to the increasing prevalence of obesity and gastroesophageal reflux disease [3,4,5]. Despite advancements in surgery, chemotherapy, and immunotherapy, the prognosis for EAC remains poor, with a five-year survival rate of 22% [6]. Additionally, advanced EAC frequently metastasizes to distant organs, with the liver being the most common site, followed by lung and bone; lymph-node, peritoneal and (rarely) brain metastases have also been reported, contributing to the poor prognosis of metastatic disease [7,8,9]. Tumor hypoxia, a hallmark of solid tumors, drives aggressive behavior and therapeutic resistance through stabilization of hypoxia-inducible factor 1-alpha (HIF1α), which regulates genes involved in angiogenesis, metabolism, and immune evasion [10,11].

Among the downstream targets of HIF1α, NT5E (CD73), an ecto-5′-nucleotidase, plays a pivotal role in the tumor microenvironment by converting extracellular AMP to immunosuppressive adenosine. NT5E-mediated adenosine accumulation suppresses anti-tumor immune responses, promotes angiogenesis, and facilitates metastasis, contributing to treatment resistance and poor clinical outcomes [12,13]. NT5E is frequently overexpressed, and its high expression is associated with advanced disease stage and poor survival across multiple cancers, including EAC [14,15,16]. Therapeutic targeting of CD73 has shown promise in preclinical models, particularly when combined with immune checkpoint inhibitors [17,18]. However, its specific role in hypoxic EAC and the direct effects of NT5E inhibition in cancer cells, independent of tumor microenvironmental influences, remain poorly understood.

In parallel, HIF1α has been implicated in regulating CD73 expression under hypoxia, linking hypoxic adaptation to adenosine-mediated immunosuppression [15,19,20,21]. However, strategies that simultaneously disrupt hypoxia-driven transcriptional programs and CD73-mediated extracellular adenosine signaling in EAC have not been fully explored. We hypothesize that dual targeting of HIF1α and CD73 will synergistically reduce EAC cell viability and disrupt pro-tumor microenvironmental pathways, offering a novel therapeutic approach for hypoxic, treatment-resistant EAC.

In this study, we investigated the regulation of NT5E by HIF1α in EAC under hypoxia, assessed the effects of dual inhibition on purinergic metabolism and cell viability, and evaluated their combined impact on migration, angiogenesis, and the extracellular microenvironment, aiming to inform the development of rational combinatorial strategies for EAC therapy.

## 2. Methods

### 2.1. Gene Expression and Survival Analysis

Gene expression data from the Cancer Cell Line Encyclopedia (CCLE) was downloaded from the DepMap portal [22] (DepMap Public 24Q2) and TCGA-ESCA data for esophageal cancer patients was downloaded from cBioPortal [23]. For stratifying patients by high and low expression of NT5E, patients were classified into two expression groups by maximally separated Kaplan–Meier plots.

### 2.2. Immunohistochemistry

Formalin-fixed paraffin-embedded (FFPE) tissue sections (4 µm) from EAC tumor and matched adjacent normal tumor were previously collected from patients being evaluated at the Goshen Center for Cancer Care in Goshen, IN [24]. Sections were baked (60 °C, 1 h), deparaffinized, rehydrated, and subjected to heat-induced epitope retrieval (10 mM Sodium Citrate pH 6.0; HIF1α: Tris-EDTA pH 9.0; 95–100 °C, 30 min). Endogenous peroxidase was quenched (3% H_2_O_2_, 10 min) and slides were blocked (5% normal horse serum, 30 min). Primary antibodies were applied overnight at 4 °C; NT5E/CD73 (D7F9A) Rabbit mAb (#13160, 1:200, Cell Signaling Technology, Danvers, MA, USA), HIF1α (D1S7W) XP^®^ Rabbit mAb (#36169, 1:100, Cell Signaling Technology, Danvers, MA, USA). Detection used an HRP-polymer secondary antibody with DAB chromogen (Vector Laboratories, Newark, CA, USA), hematoxylin counterstain (Sigma-Aldrich, St. Louis, MO, USA), dehydration, and resin mounting. H&E staining was performed per standard protocols. Negative controls (no primary/isotype) and vendor-recommended positive controls were included in each run. Brightfield images were acquired on the same microscope system under matched exposure settings.

### 2.3. Cell Culture and Hypoxia Treatment

FLO-1, OE19, and OE33 were chosen as molecularly distinct, well-characterized EAC models that exhibit measurable basal and hypoxia-induced NT5E and HIF1α expression, enabling consistent evaluation of hypoxia-responsive pathways and dual-target inhibition. FLO-1 (Cat No. 11012001-1VL), OE19 (Cat No. 96071721-1VL) and OE33 (Cat No. 96070808-1VL) cell lines were purchased from Sigma-Aldrich (St. Louis, MO, USA). Cells were cultured in RPMI 1640 supplemented with 10% fetal bovine serum and 1% penicillin-streptomycin at 37 °C and 5% CO_2_. For hypoxia experiments, cells were serum-starved (RPMI + 1% FBS) for 8 h prior to assays and were placed in a hypoxia chamber (1% O_2_, 5% CO_2_, 94% N_2_).

### 2.4. Western Blotting

Cells were lysed in RIPA buffer (Thermo Fisher Scientific, Waltham, MA, USA) supplemented with protease and phosphatase inhibitor cocktails (Roche, Indianapolis, IN, USA). Lysates were incubated on ice for 30 min, centrifuged at 14,000× *g* for 15 min at 4 °C, and supernatants were collected. Protein concentration was determined using the Pierce BCA Protein Assay Kit (Thermo Fisher Scientific, Waltham, MA, USA).

Equal amounts of protein (20 µg) were separated by SDS–PAGE using 10% Bis-Tris gels (Bio-Rad, Hercules, CA, USA) and transferred onto PVDF membranes (MilliporeSigma, Burlington, MA, USA). Membranes were blocked in 5% non-fat dry milk in TBS-T (20 mM Tris-HCl, 150 mM NaCl, 0.1% Tween-20, pH 7.6) for 1 h at room temperature and probed overnight at 4 °C with primary antibodies; HIF-1α (D1S7W) XP^®^ Rabbit mAb, #36169S, Cell Signaling Technology (Danvers, MA, USA), 1:1000; NT5E/CD73 (D7F9A) Rabbit mAb, #13160S, Cell Signaling Technology (Danvers, MA, USA), 1:1000; GAPDH (14C10) Rabbit mAb, #9043, Cell Signaling Technology (Danvers, MA, USA), 1:2000.

After washing, membranes were incubated with HRP-conjugated secondary antibodies (SouthernBiotech, Birmingham, AL, USA) for 1 h at room temperature. Bands were visualized using ECL substrate (Amersham, Buckinghamshire, UK) and imaged using the ChemiDoc Imaging System (Bio-Rad, Hercules, CA, USA). Densitometry performed using Bio-Rad’s Image Lab software (v6.1).

### 2.5. siRNA Knockdown

Cells were transfected using Trilencer-27 siRNA kits (Origene, Rockville, MD, USA) targeting NT5E, HIF1α, or non-targeting control siRNA using SiTran2.0 transfection reagent according to the manufacturer’s protocol. Knockdown efficiency was confirmed by qPCR.

### 2.6. Drug Treatments

Cells were treated with acriflavine (Sigma-Aldrich, St. Louis, MO, USA) as a HIF1α inhibitor and PSB12379 (MedChemExpress, Monmouth Junction, NJ, USA) as a CD73/NT5E inhibitor at the indicated concentrations under normoxic or hypoxic conditions for 24–72 h prior to viability, migration, or metabolite assays.

### 2.7. Quantitative PCR

Total RNA was extracted using the RNeasy Mini Kit (Qiagen, Hilden, Germany) and reverse-transcribed using the High-Capacity cDNA Reverse Transcription Kit (Applied Biosystems, Waltham, MA, USA). qPCR was performed using SYBR Green Master Mix (Bio-Rad, Hercules, CA, USA) on a CFX96 Real-Time PCR Detection System (Bio-Rad). Relative expression was normalized to β-actin using the ΔΔCt method.

### 2.8. Cell Viability Assays

Cell viability was assessed using the CellTiter-Glo Luminescent Cell Viability Assay (Promega, Madison, WI, USA) according to the manufacturer’s instructions. Luminescence was measured using a Cytation 5 plate reader (Agilent BioTek, Winooski, VT, USA).

### 2.9. Metabolite Measurements

Intracellular and extracellular AMP and adenosine levels were quantified using targeted LC-MS/MS as previously described [25]. Briefly, metabolites were extracted from cell pellets using 80% methanol (VWR, Radnor, PA, USA) in water, while conditioned media were extracted using a 4:1 ratio of cold methanol to media. Samples were vortexed for 15 min on a thermomixer set to 4 °C, followed by incubation at −80 °C for 1 h to precipitate proteins. After centrifugation (16,000× *g*, 15 min, 4 °C), supernatants were dried in a SpeedVac concentrator (Thermo Fisher Scientific, Waltham, MA, USA) and resuspended in mobile phase (95:5, 0.01% formic acid in water/acetonitrile).

Analytes were separated using an Agilent 1290 Infinity II UHPLC system and detected using an Agilent 6460 Triple Quadrupole Mass Spectrometer (QQQ) (Agilent Technologies, Santa Clara, CA, USA) in multiple reaction monitoring (MRM) mode. Data were analyzed using Agilent MassHunter software version 9.0. Metabolite abundance was expressed as the peak area of the primary MRM transition for each analyte.

### 2.10. Migration Assays

Cell migration was assessed using a Boyden chamber assay as described previously [26] (Corning Inc., Corning, NY, USA). Cells were seeded in serum-free medium in the upper chamber and allowed to migrate toward 10% FBS in the lower chamber under normoxic or hypoxic conditions for 24 h. Migrated cells were fixed with 4% paraformaldehyde, stained with crystal violet (Sigma-Aldrich, St. Louis, MO, USA), and counted.

### 2.11. Angiogenesis Assays

Endothelial tube formation assays were performed using human umbilical vein endothelial cells (HUVECs) seeded on Matrigel Basement Membrane Matrix (Corning, Corning, NY, USA), following our previously published protocol [27]. Tube formation was imaged after 8 h, and total tube length was quantified using ImageJ v1.54.

### 2.12. VEGF ELISA

Conditioned media from treated FLO-1 cells under hypoxia were analyzed for VEGF concentrations using the Human VEGF ELISA Kit (PeproTech, Rocky Hill, NJ, USA) according to the manufacturer’s instructions.

### 2.13. Statistical Analysis

Data are presented as mean ± SD. Statistical significance was determined using unpaired Student’s *t*-tests and one-way or two-way ANOVA as appropriate, using GraphPad Prism 10. *p*-values < 0.05 were considered statistically significant.

## 3. Results

### 3.1. NT5E Is Upregulated by HIF1a in Esophageal Adenocarcinoma and Negatively Correlated with Survival in Patients

HIF1a signaling has previously been shown to promote metastasis in esophageal cancer [21] and NT5E a well-known target of HIF1a signaling in epithelial cells of other cancers [15,19,20] (Supplementary Table S1) [28], therefore we interrogated the relationship of HIF1a and NT5E regarding its expression and impact on survival in esophageal cancers.

**Figure 1:**
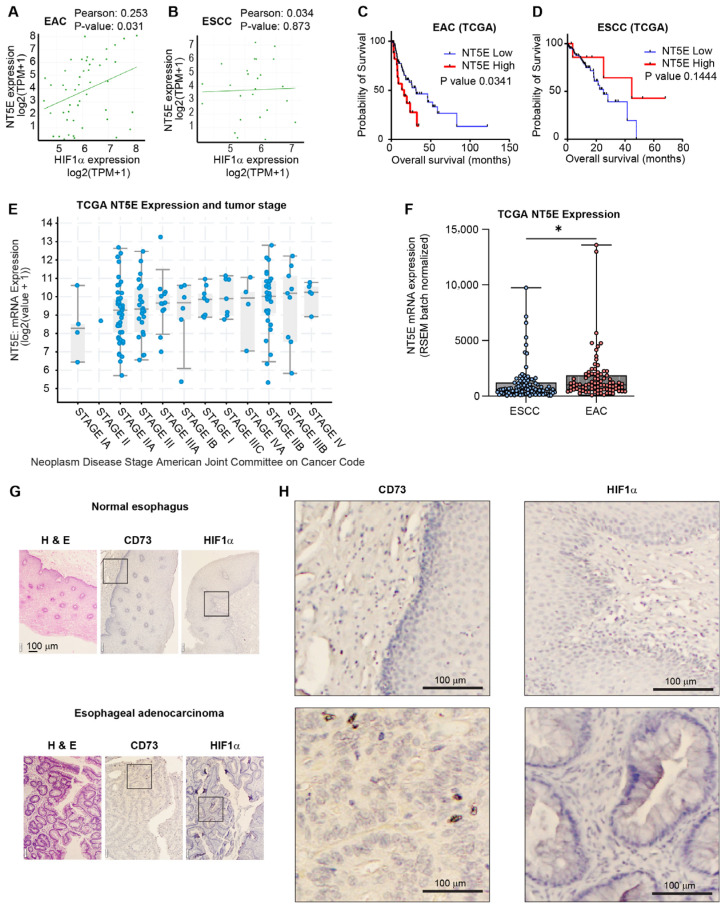
NT5E and HIF1α co-expressed in esophageal adenocarcinoma and negatively correlated with patient survival. (**A**,**B**) X/Y plot and correlation analysis between NT5E and HIF1α expression data across esophageal adenocarcinoma (EAC) cell lines (**A**) and esophageal squamous cell carcinoma (ESCC) lines (**B**) from DepMap. (**C**,**D**) Kaplan–Meier plots of EAC (**C**) and ESCC (**D**) patients stratified by NT5E expression; assessed using log-rank (Mantel–Cox) test. (**E**) NT5E expression across stages of esophageal cancer. (**F**) NT5E expression comparison between EAC and ESCC patients; significance determined by unpaired two-tailed *t*-test; * *p*-value < 0.05. (**G**) IHC of H&E stain (**left**), IHC of CD73 (**middle**) and IHC of HIF1 (**right**), in EAC tumor tissue (**bottom**) and adjacent normal tissue (**top**). (**H**) Higher-magnification view (8× enlargement) of the region indicated in Figure 1G.

Utilizing gene expression data from the Cancer Cell Line Encyclopedia, we identified a significant correlation between HIF1A and NT5E gene expression in esophageal adenocarcinoma (EAC) cell lines (Figure 1A). The correlation between HIF1A and NT5E in EAC was among the most significant across all cancer sub-types (Supplementary Table S2); however, there was no correlation between HIF1A and NT5E in esophageal squamous cell carcinoma (ESCC) cell lines (Figure 1B).

To assess clinical relevance, we performed Kaplan–Meier survival analysis on esophageal cancer patient data from TCGA-ESCA, stratified by NT5E expression levels, which revealed that high NT5E expression is associated with significantly lower overall survival compared to low expression in EAC (Figure 1C) and not ESCC (Figure 1D). Notably, NT5E expression was higher in advanced stages of esophageal cancers (Figure 1E) and significantly higher in EAC patients compared to ESCC patients (Figure 1F). Additionally, we investigated whether prior chemotherapy exposure influenced NT5E expression. Clinical annotation for chemotherapy exposure was available for only a subset of TCGA-ESCA patients, as treatment timelines were provided exclusively for individuals who received documented therapies. Patients without recorded timelines could not be confidently classified as chemotherapy-naïve, since absence of documentation does not necessarily indicate absence of treatment. Within this limited subset, we compared NT5E expression between patients recorded as having received treatment and those lacking treatment annotation. NT5E expression was modestly higher in the unannotated group; however, given the incomplete records and ambiguity regarding true treatment status, this difference should be interpreted with caution and does not support a definitive relationship between chemotherapy exposure and NT5E expression (Supplementary Figure S1). Furthermore, we performed IHCs for CD73/NT5E, and HIF1a expression in EAC patient samples, confirming high expression of both relative to non-cancerous tissue (Figure 1G,H). NT5E is associated with invasive cancer phenotypes [29] and taken together, these results highlight the importance of NT5E, and its correlation with HIF1a, specifically in EAC tumor progression, which is known to be more invasive than ESCC [30].

### 3.2. NT5E Is Transcriptionally Upregulated by HIF1α Under Hypoxic Conditions in EAC

**Figure 2:**
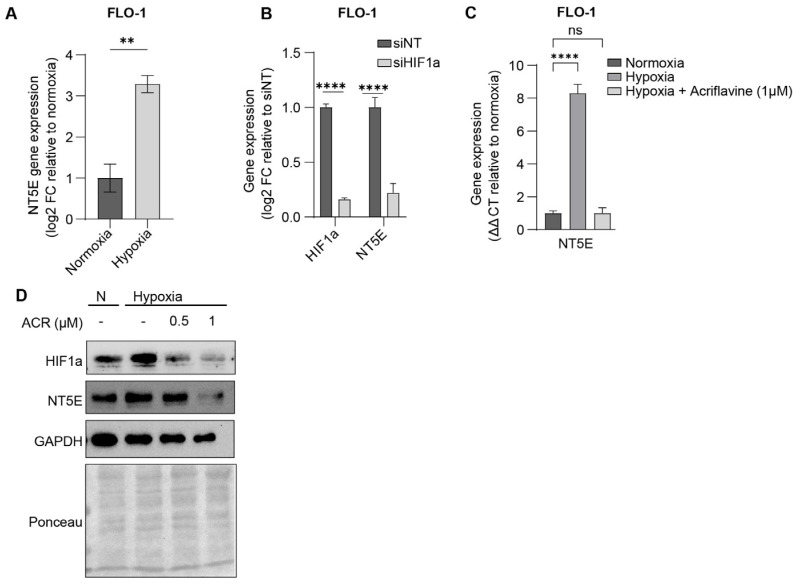
NT5E is transcriptionally upregulated by HIF1α under hypoxic conditions in EAC. (**A**) NT5E expression by qPCR in FLO-1 cells under normoxia and hypoxia; data represent mean ± SEM (*n* = 4; ** *p* < 0.01; **** *p* < 0.0001); significance determined by unpaired two-tailed *t*-test. (**B**) Effect of HIF1α knockdown on NT5E expression under hypoxia; comparisons analyzed by two-way ANOVA with Sidak correction. (**C**) Measurement of HIF1α and NT5E in FLO-1 cells treated with acriflavine by qPCR; data represent mean ± SEM (*n* = 4); analyzed by one-way ANOVA with Dunnett’s correction for multiple comparisons. (**D**) Western blot confirmation of NT5E protein regulation under hypoxia with acriflavine treatment. Uncropped images and densitometry appended to Supplementary Material document after Supplementary Figure S5.

To determine if HIF1α regulates NT5E expression in EAC, we exposed EAC cells to hypoxic conditions and observed significant upregulation of NT5E expression in multiple cell lines by qPCR (Figure 2A, and Supplementary Figure S2A,B). The hypoxia induced regulatory activity of HIF1a was verified by siRNA-mediated knockdown of HIF1a by qPCR (Figure 2B). Acriflavine, a known HIF1a inhibitor, also attenuated the hypoxia-induced expression of NT5E, measured by qPCR (Figure 2C and Supplementary Figure S2C), and Western blot (Figure 2D and Supplementary Figure S2D,E) confirming that HIF1a activity is essential for NT5E upregulation. These findings highlight the importance of NT5E on tumor progression and the regulatory role of HIF1a in EAC.

### 3.3. EAC Cells Are Sensitive to HIF1α and NT5E Inhibition, with Enhanced Effects Under Hypoxic Conditions

**Figure 3:**
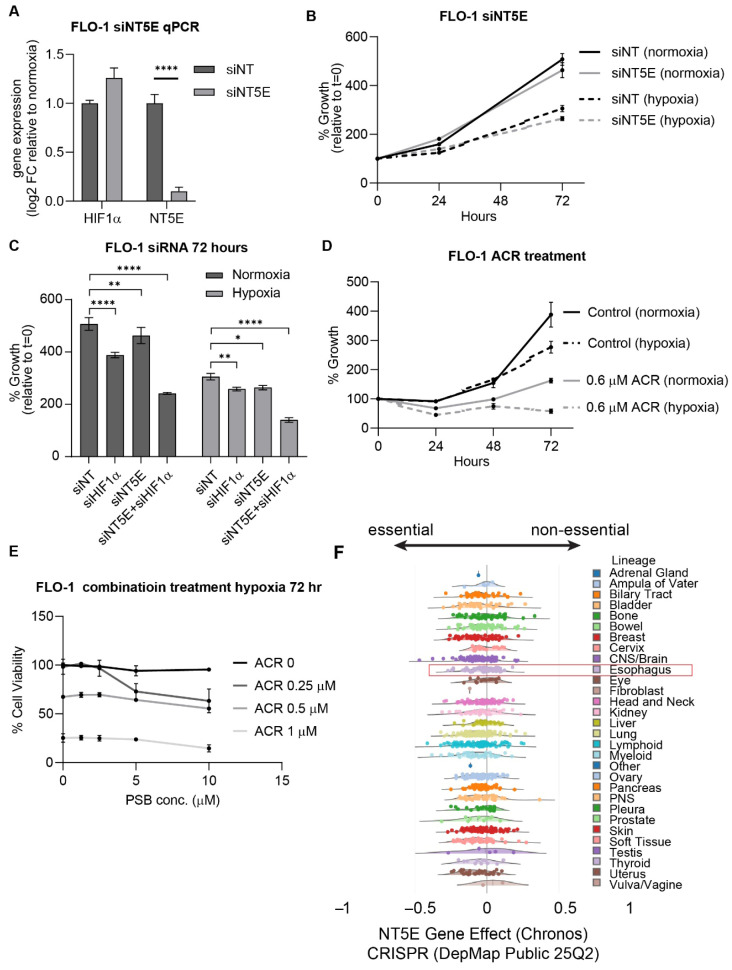
EAC cells are sensitive to HIF1α and NT5E inhibition, with enhanced effects under hypoxic conditions. (**A**,**B**) HIF1α and NT5E expression measured by qPCR (**A**) and cell viability measurements expressed as % growth (cell titer relative to day 0) (**B**) upon NT5E knockdown; qPCR data represent mean ± SEM of *n* = 4; statistical significance determined by unpaired two-tailed *t*-test. (**C**) Cell viability in HIF1α knockdown alone and in combination with NT5E knockdown under normoxic or hypoxic conditions at 72 h; data are mean ± SEM (*n* = 3); comparisons analyzed by two-way ANOVA with Dunnet’s correction. (**D**) % cell growth (cell titer relative to day 0) comparing acriflavine treatment in normoxic and hypoxic conditions. (**E**) % cell growth for combined treatment with acriflavine and PSB12379 under normoxic and hypoxic conditions, showing synergistic viability reduction. (**F**) NT5E CRISPR dependency scores from DepMap across cancer lineages, indicating NT5E is broadly non-essential. ns = not significant; * *p*-value < 0.05; ** *p* < 0.01; **** *p* < 0.0001.

To evaluate the therapeutic relevance of NT5E and HIF1α, we performed siRNA-mediated knockdown experiments in FLO-1 esophageal adenocarcinoma (EAC) cells. Silencing of NT5E modestly decreased cell proliferation (Figure 3A,B). Knockdown of HIF1α significantly reduced cell viability, which was further amplified under hypoxic conditions, and dual knockdown of HIF1α and NT5E under hypoxia further decreased cell growth compared to single knockdowns (Figure 3C).

To complement genetic knockdown approaches, we treated cells with the HIF1α inhibitor acriflavine and the NT5E inhibitor PSB12379. Acriflavine markedly suppressed FLO-1 cell growth, and its potency increased under hypoxia (Figure 3D and Supplementary Figure S3A,B). PSB12379 alone exhibited minimal growth inhibition; however, combination treatment with acriflavine and PSB12379 resulted in synergistic growth suppression under hypoxic conditions, even at concentrations below the acriflavine IC50 (Figure 3E and Supplementary Figure S3C).

To contextualize these findings, we examined NT5E CRISPR dependency scores in the DepMap portal, which revealed that NT5E is broadly non-essential across cancer lineages, including EAC cell lines (Figure 3F). This observation aligns with our findings that NT5E knockdown or PSB12379 treatment alone has only modest effects on EAC cell growth, while combined inhibition with HIF1α, particularly under hypoxic conditions, significantly reduces cell viability. These results revealed a context-dependent therapeutic vulnerability to NT5E inhibition, and highlight the potential of dual targeting to overcome the limited single-agent efficacy of NT5E inhibition by leveraging the hypoxia-driven dependency on HIF1α pathways.

### 3.4. HIF1α and NT5E Inhibition Alters Purinergic Metabolite Levels in EAC Cells

**Figure 4:**
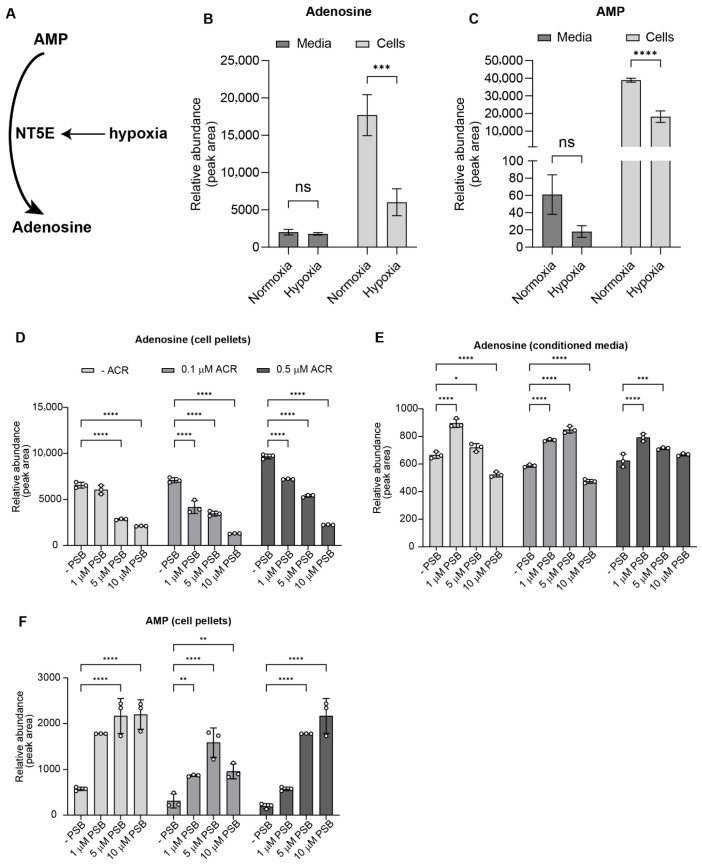
HIF1α and NT5E inhibition alter purinergic metabolite levels in esophageal adenocarcinoma cells. (**A**) Schematic illustrating hypoxia-induced NT5E expression and the enzymatic conversion of AMP to adenosine. (**B**) Intracellular and extracellular adenosine levels in FLO-1 cells measured by LC-MS, cultured under normoxia or hypoxia. Data represent mean ± SD (*n* = 3 biological replicates); significance determined by unpaired two-tailed *t*-test. (**C**) Intracellular and extracellular AMP levels under the same conditions. Data represent mean ± SD (*n* = 3); significance determined by unpaired two-tailed *t*-test. (**D**) Dose-dependent changes in intracellular adenosine following treatment with PSB12379, acriflavine, or their combination under hypoxia. Data represent mean ± SD (*n* = 3); analyzed by two-way ANOVA with Tukey’s multiple comparison test. (**E**) Adenosine levels in conditioned media from the same experiment, showing dose-dependent accumulation at low and intermediate PSB12379 concentrations, followed by reduction at the highest dose. Data represent mean ± SD (*n* = 3); analyzed by two-way ANOVA with Tukey’s post hoc test. (**F**) Intracellular AMP levels under the same treatment conditions; PSB12379-induced AMP accumulation further enhanced by acriflavine co-treatment. Data represent mean ± SD (*n* = 3); analyzed by two-way ANOVA with Tukey’s multiple comparison test. ns = not significant; * *p* < 0.05; ** *p* < 0.01; *** *p* < 0.001; **** *p* < 0.0001.

To examine how hypoxia and inhibition of HIF1α or NT5E influence purine metabolism, we quantified intracellular and extracellular levels of adenosine monophosphate (AMP) and adenosine in FLO-1 esophageal adenocarcinoma (EAC) cells. A schematic depicting hypoxia-induced NT5E expression and its enzymatic conversion of AMP to adenosine is shown in Figure 4A.

Under hypoxia, intracellular adenosine levels decreased unexpectedly, while extracellular adenosine levels remained largely unchanged (Figure 4B). Consistent with NT5E enzymatic activity, intracellular AMP levels were reduced under hypoxia, with only marginal decreases in the media (Figure 4C), suggesting our earlier finding that hypoxia-induced NT5E expression is linked to increased AMP-to-adenosine turnover.

To determine whether these effects depend on NT5E function, FLO-1 cells were treated with increasing concentrations of the NT5E inhibitor PSB12379, the HIF1α inhibitor acriflavine, or their combination under hypoxia. PSB12379 induced a dose-dependent decrease in intracellular adenosine (Figure 4D) and a corresponding increase in adenosine levels in conditioned media up to intermediate concentrations (Figure 4E), followed by a decline at the highest dose, consistent with partial inhibition of extracellular adenosine turnover. In contrast, acriflavine treatment alone elevated intracellular adenosine, likely reflecting broader effects of HIF1α inhibition on purine metabolism. Notably, combined treatment with acriflavine and PSB12379 still resulted in a net decrease in intracellular adenosine, indicating that NT5E inhibition predominates over the opposing effect of acriflavine.

Analysis of AMP levels revealed a dose-dependent accumulation in PSB12379-treated cells, consistent with effective blockade of NT5E catalytic activity (Figure 4F). This effect was exacerbated by co-treatment with acriflavine, suggesting that inhibition of HIF1α amplifies AMP buildup, potentially through suppression of compensatory metabolic pathways. Similar increases in the intracellular AMP/adenosine ratio were observed in additional EAC cell lines (Supplementary Figure S4A), confirming the generalizability of this effect.

Collectively, these data indicate that hypoxia enhances AMP turnover through NT5E, and that dual inhibition of HIF1α and NT5E disrupts purine homeostasis, leading to accumulation of upstream metabolites and depletion of adenosine pools. These metabolic perturbations likely contribute to the observed synergistic reduction in cell viability under dual treatment conditions.

### 3.5. Dual Inhibition of HIF1α and NT5E Reduces Migration and Pro-Angiogenic Signaling in EAC Cells

**Figure 5:**
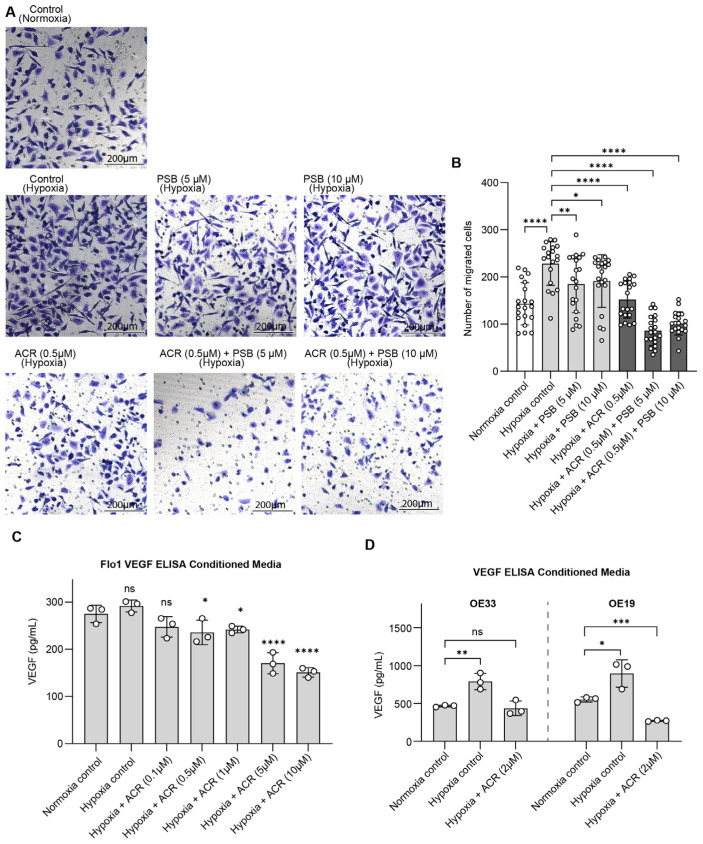
Dual inhibition of HIF1α and NT5E disrupts extracellular pathways associated with immune evasion and metastasis. (**A**,**B**) Representative images (**A**) and cell counts (**B**) in FLO-1 for the cell migration assay under hypoxia with indicated treatments; data represent mean ± SD of cell counts for *n* = 20 images across 2 separate experiments, analyzed by one-way ANOVA. (**C**,**D**) VEGF levels in conditioned media under hypoxia with indicated treatments, data represent mean ± SD (*n* = 3) analyzed by one-way ANOVA. ns = not significant; * *p* < 0.05; ** *p* < 0.01; *** *p* < 0.001; **** *p* < 0.0001.

While NT5E’s role in supporting EAC cell viability under hypoxia highlights its tumor-intrinsic functions, NT5E is best characterized for its contributions to the tumor microenvironment, where CD73-generated adenosine promotes immune evasion, angiogenesis, and metastatic potential. To investigate these extracellular consequences, we next performed cell migration assays, demonstrating that combined HIF1α and NT5E inhibition significantly reduced FLO-1 cell migration under hypoxia relative to single-agent treatment, indicating that dual targeting can attenuate cellular behaviors associated with invasion and metastasis (Figure 5A,B).

To investigate microenvironmental factors that support metastatic potential, angiogenesis assays were performed using endothelial tube formation assays with HUVECs in conditioned media collected from EAC cells treated with acriflavine, PSB12379, or the combination under hypoxia. Conditioned media from dual-treated cells reduced endothelial tube formation compared to single treatments or controls, indicating inhibited pro-angiogenic signaling (Supplementary Figure S5A).

To directly assess the effect of HIF1α and NT5E inhibition on VEGF secretion, a key pro-angiogenic factor, we performed VEGF ELISAs in conditioned media collected from EAC cells treated with acriflavine, PSB12379 and their combination. VEGF levels were elevated in EAC cell lines under hypoxic conditions and reduced by acriflavine treatment, indicating that HIF1α-driven pro-angiogenic signaling is effectively attenuated (Figure 5C,D). Notably, PSB12379 did not reduce VEGF levels, and in the combination treatment group, levels were reduced similarly to acriflavine alone (Supplementary Figure S5B). This suggests that while NT5E inhibition does not directly impact VEGF production, it may enhance the overall anti-tumor microenvironmental effects of HIF1α inhibition when used in combination.

Together, these results demonstrate that dual targeting of HIF1α and NT5E in EAC cells not only impacts tumor cell viability but also disrupts key extracellular pathways driving immune evasion, angiogenesis, and metastatic potential, underscoring the multifaceted therapeutic potential of this combinatorial approach in esophageal adenocarcinoma.

## 4. Discussion

Esophageal adenocarcinoma (EAC) remains difficult to treat, particularly within tumor hypoxic niches that stabilize HIF1α and drive therapeutic resistance and metastasis [21,31,32,33]. In this study, we identify NT5E/CD73 as a direct hypoxia-responsive effector in EAC that is transcriptionally regulated by HIF1α, and we demonstrate that simultaneous disruption of HIF1α and NT5E suppresses tumor cell viability and perturbs key extracellular programs linked to immune evasion, angiogenesis, and migration. Consistent with previous reports of CD73 as a mediator of adenosine-driven immunosuppression and poor outcomes in multiple cancers [15,19,20], we found that NT5E expression correlates with HIF1α specifically in EAC but not in ESCC, associates with reduced overall survival, and increases with disease progression, establishing NT5E as a clinically relevant, EAC-specific vulnerability.

Mechanistically, we found that hypoxia robustly induces NT5E expression in EAC cells through HIF1α, as confirmed by siRNA knockdown and pharmacological inhibition with acriflavine. This induction is functionally significant, since dual inhibition of HIF1α and NT5E under hypoxia produced synergistic suppression of cell viability, revealing a cooperative dependence on these two pathways for EAC survival. Interestingly, CRISPR dependency profiling across cancer lineages indicated that NT5E is broadly non-essential under normoxia, consistent with the limited effects of NT5E inhibition alone on EAC cell growth. However, under hypoxic conditions where HIF1α drives survival pathways, NT5E inhibition becomes highly impactful in combination, suggesting a context-dependent therapeutic vulnerability that could be therapeutically exploited in hypoxic tumors. Although acriflavine and PSB12379 are primarily research tools rather than clinical-stage therapeutics, their mechanisms highlight pathways already being targeted in ongoing drug development efforts. Notably, clinically advanced CD73 inhibitors such as oleclumab (MEDI9447) are in Phase I/II trials for solid tumors [34,35], supporting the translational relevance of CD73 blockade and underscoring the potential for more selective HIF1α and CD73 inhibitors to be incorporated into future combinatorial strategies for EAC.

Our LC/MS data further illuminate this mechanism. Under hypoxia, intracellular adenosine and AMP both decreased, suggesting enhanced AMP turnover and dynamic adenosine trafficking rather than simple accumulation. One plausible explanation is transport dominance: hypoxia can upregulate equilibrative nucleoside transporters (ENT1/ENT2), favoring adenosine efflux and altering re-uptake kinetics in a pH- and membrane-potential–dependent manner [36,37]. Concurrently, adenosine may be rapidly catabolized—extracellularly by ADA to inosine and intracellularly by ADK to AMP—thereby maintaining low steady-state adenosine without requiring large changes in total nucleotide pools [38,39]. These multilayered feedbacks, combined with metabolic shifts in glycolysis and purine synthesis under hypoxia [40,41], provide a coherent explanation for the observed AMP–adenosine balance.

Pharmacologic perturbations support this view. PSB12379 produced dose-dependent intracellular AMP accumulation consistent with on-target NT5E inhibition, while intracellular adenosine decreased. In conditioned media, adenosine increased at low–intermediate PSB concentrations and declined at the highest dose (Figure 4E), suggesting a biphasic balance: partial NT5E inhibition may increase transient extracellular adenosine via altered release/re-uptake or enzyme/substrate saturation, whereas stronger inhibition limits net production from AMP. Notably, acriflavine alone increased intracellular adenosine (Figure 4D), consistent with broader HIF1α program suppression (e.g., reduced ADA and ENT activity, or altered purine salvage) rather than direct NT5E effects. Despite this, PSB12379 overrode this increase when combined, indicating NT5E’s gatekeeping role for adenosine under hypoxia while other metabolic alterations are in play. The AMP/adenosine ratio shifts we observed across additional EAC lines demonstrate that these flux changes are generalizable.

Taken together, these mechanisms provide a coherent framework for the observed drop in intracellular adenosine despite hypoxia-induced NT5E, and they help explain the dose- and compartment-specific responses to PSB12379 and acriflavine in Figure 4. Future studies will include a more comprehensive, targeted metabolomics investigation with isotope tracing, paired RNA-seq, and integrated gene–metabolite network analyses across hypoxic perturbations (HIF1α/NT5E single and dual inhibition). This approach would precisely map flux control points and pathway crosstalk with higher precision. Additionally, a multi-omic dissection may uncover novel biochemical circuits and basic cellular principles of purine homeostasis under hypoxic stress beyond the therapeutic implications we explored here.

Functionally, dual treatment also inhibited cell migration, HUVEC tube formation and VEGF secretion by tumor cells under hypoxia via HIF1α blockade, aligning with canonical HIF1α-VEGF regulation [31,33]. While NT5E inhibition alone did not suppress VEGF production, it likely attenuates adenosine-mediated paracrine signaling that sustains angiogenesis and immune evasion [42,43,44]. Together, these findings support a two-axis mechanistic model: (i) HIF1α inhibition disrupts transcriptional programs controlling hypoxic survival and angiogenesis, and (ii) NT5E inhibition limits extracellular adenosine accumulation and downstream purinergic signaling. Their convergence yields synergistic suppression of both tumor-intrinsic and microenvironmental pro-tumor pathways in EAC.

We acknowledge certain limitations. The current study was confined to in vitro models that do not fully recapitulate stromal and immune complexity in the tumor microenvironment. Future studies using 3D organoid or in vivo xenograft models of EAC will be essential to determine the physiological relevance of dual (HIF1α and NT5E) inhibition on tumor growth, angiogenesis, immune modulation and metastasis. Moreover, while acriflavine was used as a pharmacological HIF1α inhibitor, its broad action may extend beyond HIF1α; thus, employing genetic silencing or more selective small-molecule inhibitors would further strengthen mechanistic precision. Additionally, future studies will investigate the effect of NT5E/CD73 inhibition within immunomodulatory contexts using 3D organoids and co-culture systems incorporating macrophages or T cells. Lastly, while increased CD73 expression has been associated with enhanced migratory potential in prior studies, in vivo systems will be required to fully define the physiological relevance of CD73 overexpression independent of HIF1α in EAC.

## 5. Conclusions

In summary, our findings establish that concurrent targeting of HIF1α and CD73 offers a mechanistically integrated, multi-pathway therapeutic strategy for esophageal adenocarcinoma. This approach not only impairs hypoxia-driven transcriptional survival programs but also neutralizes adenosine-mediated microenvironmental adaptation, providing a strong biological rationale for developing combinatorial therapies for hypoxic, treatment-resistant EAC.

## Data Availability

Public datasets used in this study include DepMap and TCGA. Raw and processed data for all figures/analyses are available from the corresponding author upon reasonable request.

## Supplementary Materials

The following supporting information can be downloaded at https://www.mdpi.com/article/10.3390/cancers17244016/s1, Table S1: HIF1α Transcription targets (from PubChem pathway ID: 40791 hif1_tfpathway). Table S2: Correlation between HIF1A and NT5E gene expression in the Cancer Cell Line Encyclopedia by cancer lineage (DepMap portal). Figure S1: NT5E expression in TCGA-ESCA patients stratified by presence or absence of documented chemotherapy, related to Figure 1. Figure S2: Expanded validation of hypoxia-induced NT5E regulation in EAC cell lines, related to Figure 2. Figure S3: Additional viability analyses with acriflavine and PSB12379 treatments, related to Figure 3. Figure S4: AMP / Adenosine ratio measured by LC-MS in additional EAC lines, related to Figure 4. Figure S5: Effect of dual HIF1α and NT5E inhibition on tumor microenvironment-associated angiogenic pathways, related to Figure 5.
