# Supplementary material for: Synergistic Disruption of Survival and Metastatic Potential in Esophageal Adenocarcinoma Cells Through Combined Inhibition of HIF1α and CD73
Source: Cancers (Basel). 2025 Dec 17;17(24):4016. doi: 10.3390/cancers17244016 (PMC12730838; doi:10.3390/cancers17244016)

Synergistic Disruption of Survival and Metastatic Potential in Esophageal Adenocarcinoma Cells through Combined Inhibition of HIF1α and CD73

SUPPLEMENTARY MATERIALS

Supplementary Table S1

HIF1α Transcription targets (from PubChem pathway ID: 40791 hif1_tfpathway)

Gene id	Gene symbol	Gene name
123	PLIN2	perilipin 2
133	ADM	adrenomedullin
207	AKT1	AKT serine/threonine kinase 1
226	ALDOA	aldolase, fructose-bisphosphate A
405	ARNT	aryl hydrocarbon receptor nuclear translocator
664	BNIP3	BCL2 interacting protein 3
768	CA9	carbonic anhydrase 9
1356	CP	ceruloplasmin
1385	CREB1	cAMP responsive element binding protein 1
1906	EDN1	endothelin 1
2022	ENG	endoglin
2023	ENO1	enolase 1
2056	EPO	erythropoietin
2113	ETS1	ETS proto-oncogene 1, transcription factor
2235	FECH	ferrochelatase
2353	FOS	Fos proto-oncogene, AP-1 transcription factor subunit
2624	GATA2	GATA binding protein 2
2645	GCK	glucokinase
3091	HIF1A	hypoxia inducible factor 1 subunit alpha
3098	HK1	hexokinase 1
3099	HK2	hexokinase 2
3162	HMOX1	heme oxygenase 1
3172	HNF4A	hepatocyte nuclear factor 4 alpha
3398	ID2	inhibitor of DNA binding 2
3484	IGFBP1	insulin like growth factor binding protein 1
3689	ITGB2	integrin subunit beta 2
3725	JUN	Jun proto-oncogene, AP-1 transcription factor subunit
3939	LDHA	lactate dehydrogenase A
3952	LEP	leptin
4088	SMAD3	SMAD family member 3
4089	SMAD4	SMAD family member 4
4170	MCL1	MCL1 apoptosis regulator, BCL2 family member
4843	NOS2	nitric oxide synthase 2
4869	NPM1	nucleophosmin 1
4907	NT5E	5'-nucleotidase ecto
5045	FURIN	furin, paired basic amino acid cleaving enzyme
5054	SERPINE1	serpin family E member 1
5209	PFKFB3	6-phosphofructo-2-kinase/fructose-2,6-biphosphatase 3

5211	PFKL	phosphofructokinase, liver type
5230	PGK1	phosphoglycerate kinase 1
5236	PGM1	phosphoglucomutase 1
5243	ABCB1	ATP binding cassette subfamily B member 1
5315	PKM	pyruvate kinase M1/2
6095	RORA	RAR related orphan receptor A
6387	CXCL12	C-X-C motif chemokine ligand 12
6513	SLC2A1	solute carrier family 2 member 1
6515	SLC2A3	solute carrier family 2 member 3
6667	SP1	Sp1 transcription factor
7015	TERT	telomerase reverse transcriptase
7018	TF	transferrin
7033	TFF3	trefoil factor 3
7037	TFRC	transferrin receptor
7422	VEGFA	vascular endothelial growth factor A
7852	CXCR4	C-X-C motif chemokine receptor 4
8553	BHLHE40	basic helix-loop-helix family member e40
8648	NCOA1	nuclear receptor coactivator 1
9429	ABCG2	ATP binding cassette subfamily G member 2 (JR blood group)
10370	CITED2	Cbp/p300 interacting transactivator with Glu/Asp rich carboxy-terminal domain 2
10397	NDRG1	N-myc downstream regulated 1
10499	NCOA2	nuclear receptor coactivator 2
10987	COPS5	COP9 signalosome subunit 5
51564	HDAC7	histone deacetylase 7
54583	EGLN1	egl-9 family hypoxia inducible factor 1
79365	BHLHE41	basic helix-loop-helix family member e41
112399	EGLN3	egl-9 family hypoxia inducible factor 3

Supplementary Table S2

Correlation between HIF1A and NT5E gene expression in the Cancer Cell Line Encyclopedia by cancer lineage (DepMap portal)

Group	Number of cell lines	Pearson	Spearman	Slope	Intercept	p-value (linregress)
Non-Small Cell Lung Cancer	141	0.423	0.434	9.87E-01	-1.37E+00	1.71E-07
Rhabdomyosarcoma	19	0.763	0.799	2.24E+00	-1.16E+01	1.46E-04
Head and Neck Squamous Cell Carcinoma	64	0.425	0.478	9.12E-01	-1.25E+00	4.65E-04
Melanoma	87	0.364	0.318	9.20E-01	-9.97E-01	5.31E-04
Invasive Breast Carcinoma	60	0.331	0.408	7.07E-01	-1.45E+00	9.69E-03
Esophagogastric Adenocarcinoma	48	0.336	0.408	8.92E-01	-1.57E+00	1.97E-02
Breast Ductal Carcinoma In Situ	5	0.927	0.9	1.18E+00	-4.56E+00	2.36E-02
Neuroblastoma	33	0.375	0.319	1.43E+00	-6.35E+00	3.16E-02
Non-Cancerous	80	0.234	0.134	3.98E-01	4.39E+00	3.65E-02
Hepatocellular Carcinoma	23	0.428	0.392	7.50E-01	-5.26E-01	4.18E-02
Synovial Sarcoma	6	0.826	0.6	1.66E+00	-5.01E+00	4.26E-02
Embryonal Tumor	16	0.508	0.182	9.84E-01	-4.10E+00	4.47E-02
B-Lymphoblastic Leukemia/Lymphoma	25	0.402	0.355	7.21E-01	-1.97E+00	4.62E-02
Myeloproliferative Neoplasms	18	0.462	0.467	4.73E-01	-1.69E+00	5.34E-02
Ocular Melanoma	11	0.591	0.482	1.46E+00	-5.10E+00	5.57E-02
Mature B-Cell Neoplasms	93	-0.195	-0.15	-2.15E-01	1.94E+00	6.06E-02
Acute Myeloid Leukemia	43	0.279	0.372	4.73E-01	-1.41E+00	7.05E-02
Chondrosarcoma	4	0.871	0.8	1.60E+00	-5.99E+00	1.29E-01
Ovarian Epithelial Tumor	64	0.179	0.191	3.38E-01	2.12E+00	1.56E-01
T-Lymphoblastic Leukemia/Lymphoma	14	0.389	0.481	1.76E-01	-3.76E-01	1.69E-01
Nerve Sheath Tumor	7	0.575	0.321	1.28E+00	-3.58E+00	1.77E-01
Colorectal Adenocarcinoma	79	0.153	0.159	4.18E-01	1.87E+00	1.77E-01
Mature T and NK Neoplasms	21	0.295	0.402	2.70E-01	-8.37E-01	1.95E-01
Undifferentiated Pleomorphic Sarcoma/Malignant Fibrous Histiocytoma/High-Grade Spindle Cell Sarcoma	4	0.788	0.8	1.86E+00	-6.16E+00	2.12E-01
Ewing Sarcoma	22	0.266	0.043	5.07E-01	-1.84E+00	2.32E-01
Diffuse Glioma	71	0.14	0.087	3.53E-01	3.18E+00	2.45E-01
Meningothelial Tumor	4	0.751	0.8	5.54E-01	3.76E+00	2.49E-01
Bladder Urothelial Carcinoma	33	-0.201	-0.242	-4.23E-01	7.69E+00	2.62E-01
Cervical Adenocarcinoma	5	0.602	0.8	1.15E+00	-4.68E+00	2.83E-01
Cervical Squamous Cell Carcinoma	13	0.322	0.258	4.97E-01	1.17E+00	2.84E-01
Hodgkin Lymphoma	7	0.449	0.393	9.54E-01	-4.77E+00	3.12E-01
Liposarcoma	10	0.348	0.467	8.60E-01	-3.16E-01	3.24E-01

Lung Neuroendocrine Tumor	57	-0.132	-0.016	-3.62E-01	3.30E+00	3.28E-01
Uterine Sarcoma/Mesenchymal	4	0.613	0.4	2.19E+00	-9.64E+00	3.87E-01
Anaplastic Thyroid Cancer	8	-0.335	-0.405	-4.41E-01	9.21E+00	4.17E-01
Non-Seminoma Tous Germ Cell Tumor	6	0.398	0.232	1.23E-01	-2.98E-01	4.35E-01
Cutaneous Squamous Cell Carcinoma	5	-0.436	-0.6	-4.22E-01	6.70E+00	4.62E-01
Rhabdoid Cancer	13	-0.222	-0.253	-1.14E+00	7.55E+00	4.67E-01
Leiomyosarcoma	5	0.402	0	8.39E-01	6.33E-01	5.03E-01
Intraductal Papillary Neoplasm of the Bile Duct	35	-0.112	-0.139	-1.40E-01	6.18E+00	5.21E-01
Gestational Trophoblastic Disease	3	-0.658	-0.5	-4.62E-02	3.25E-01	5.43E-01
Renal Cell Carcinoma	36	0.079	0.128	8.26E-02	4.22E+00	6.48E-01
Pancreatic Adenocarcinoma	50	-0.064	-0.074	-9.64E-02	6.48E+00	6.59E-01
Well-Differentiated Thyroid Cancer	6	0.215	0.029	3.45E-01	4.26E+00	6.82E-01
Osteosarcoma	16	0.1	0.068	2.18E-01	2.99E+00	7.14E-01
Intracholecystic Papillary Neoplasm	6	-0.154	0.029	-4.26E-01	8.72E+00	7.71E-01
Prostate Adenocarcinoma	9	0.102	0.15	3.72E-01	8.15E-01	7.95E-01
Merkel Cell Carcinoma	3	-0.208	-0.5	-1.65E+00	1.79E+01	8.67E-01
Esophageal Squamous Cell Carcinoma	25	0.034	0.048	9.33E-02	3.17E+00	8.73E-01
Endometrial Carcinoma	33	0.023	0.139	5.04E-02	3.69E+00	8.98E-01
Non-Hodgkin Lymphoma	6	0.03	0.086	4.93E-02	1.77E-01	9.55E-01
Pleural Mesothelioma	22	0.009	0.047	1.81E-02	5.10E+00	9.67E-01
Ampullary Carcinoma	4	0.012	0.2	1.32E-02	6.05E+00	9.88E-01

Supplementary Figure S1

NT5E expression in TCGA-ESCA patients stratified by presence or absence of documented chemotherapy, related to Figure 1.

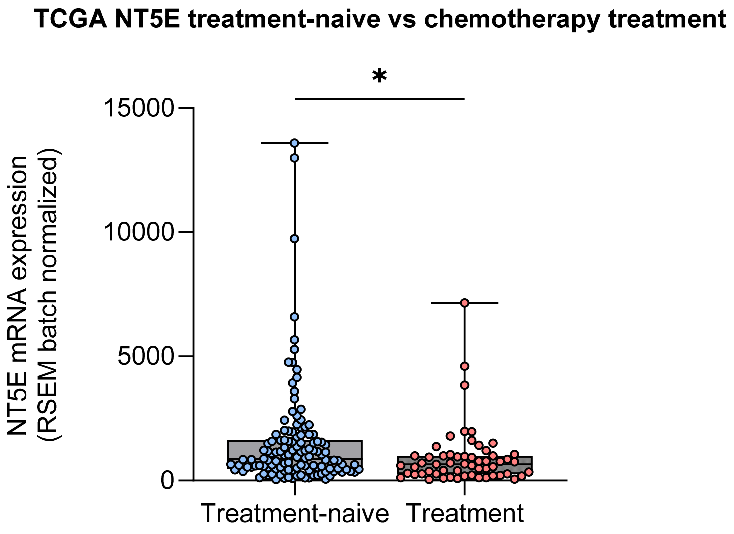

Note: absence of recorded therapy does not guarantee chemotherapy-naïve status; timelines are only available for patients with documented treatment histories.

Supplementary Figure S2

Expanded validation of hypoxia-induced NT5E regulation in EAC cell lines, related to Figure 2.

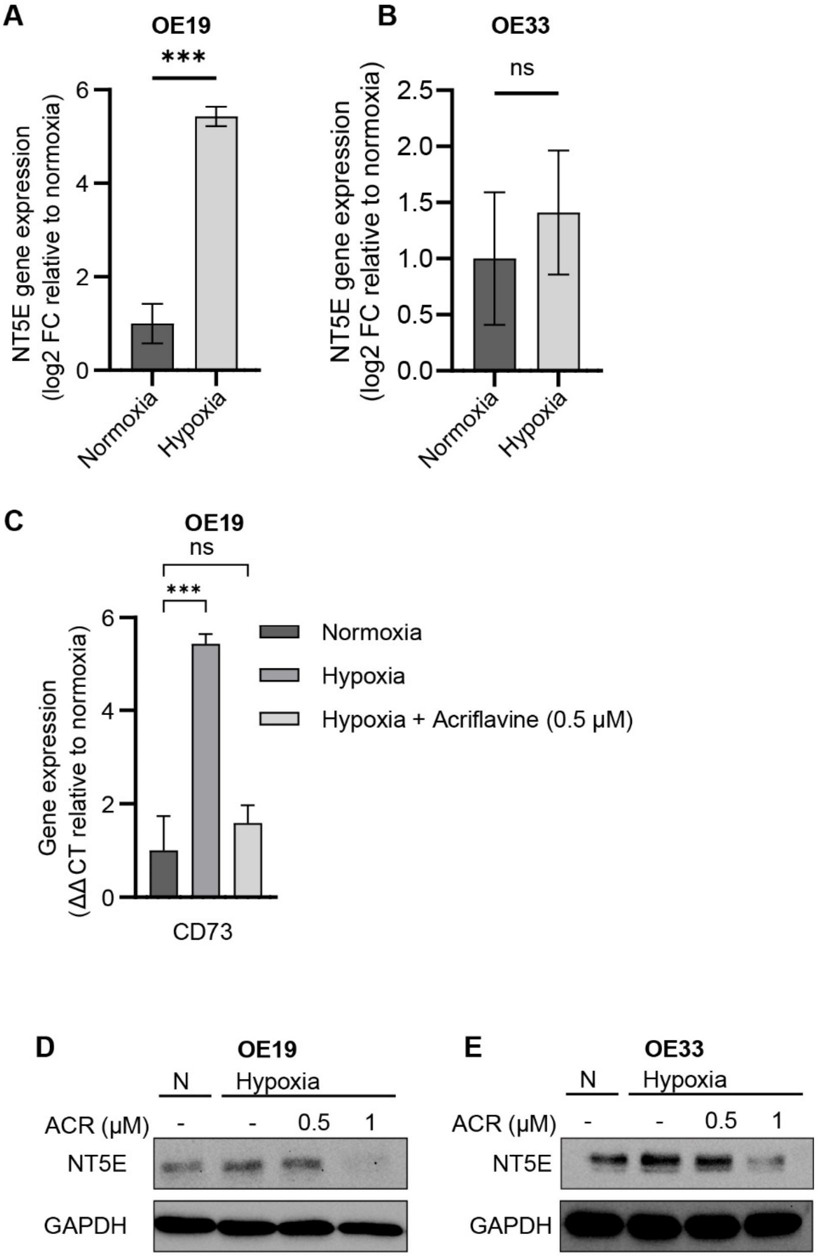

(A-B) NT5E mRNA expression under normoxic and hypoxic conditions in additional EAC cell lines (OE33, and OE19) as determined by qPCR. (C) NT5E mRNA levels under hypoxia with acriflavine treatment in additional EAC line. (D-E) Western blot validation of NT5E protein upregulation under hypoxia and its attenuation with acriflavine treatment in additional EAC lines. Uncropped images and densitometry included after Supplementary Figure S5 of this document.

Supplementary Figure S3

Additional viability analyses with acriflavine and PSB12379 treatments, related to Figure 3.

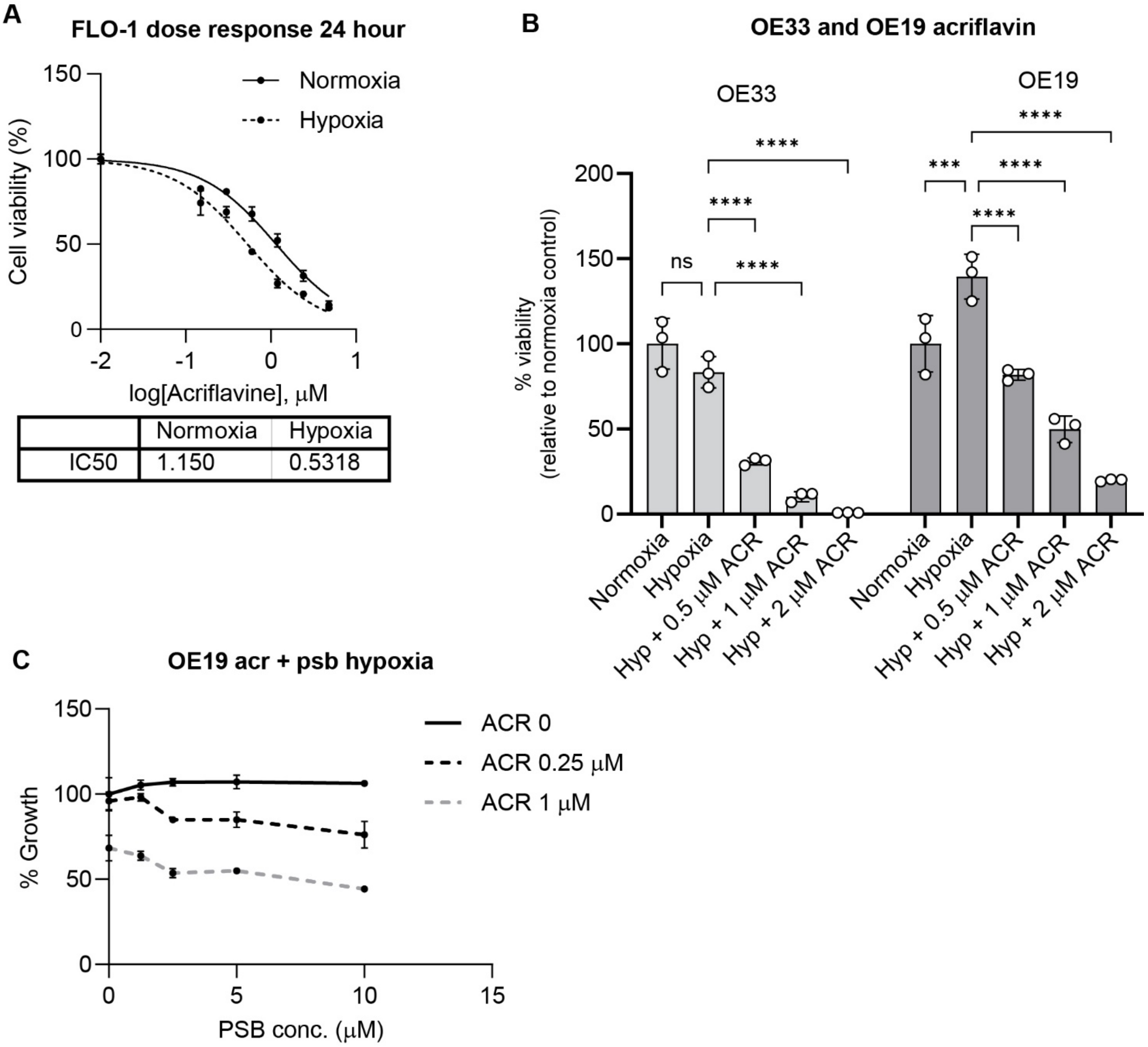

(A) Dose-response curve and IC_{50} values of acriflavine in FLO-1 cells under normoxic and hypoxic conditions after 72 hours of treatment. (B) Cell viability assays in additional EAC lines treated with acriflavine at concentrations of 0.5, 1, or 2 μM for 72 hours. (C) Combined treatment with acriflavine and PSB12379 under hypoxia in EAC line OE19.

Supplementary Figure S4

AMP / Adenosine ratio measured by LC-MS in additional EAC lines, related to Figure 4.

A

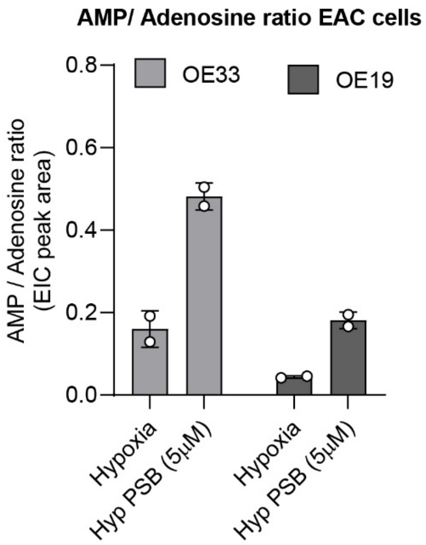

(A) AMP/Adenosine ratio in 2 additional EAC lines upon treatment with NT5E inhibition.

Supplementary Figure S5

Effect of dual HIF1α and NT5E inhibition on tumor microenvironment-associated angiogenic pathways, related to Figure 5.

A

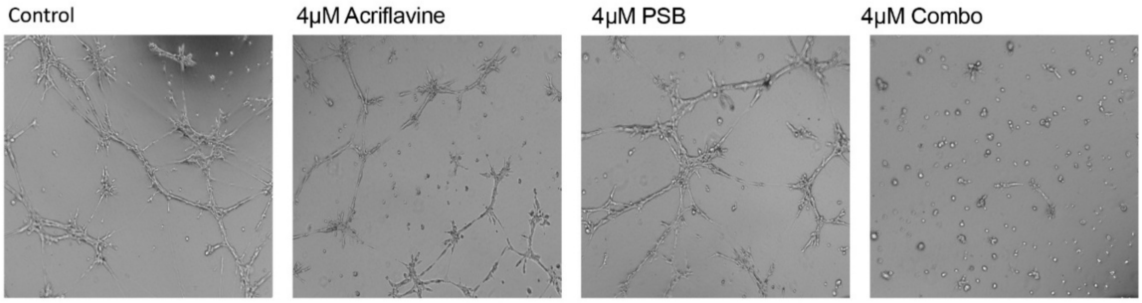

B

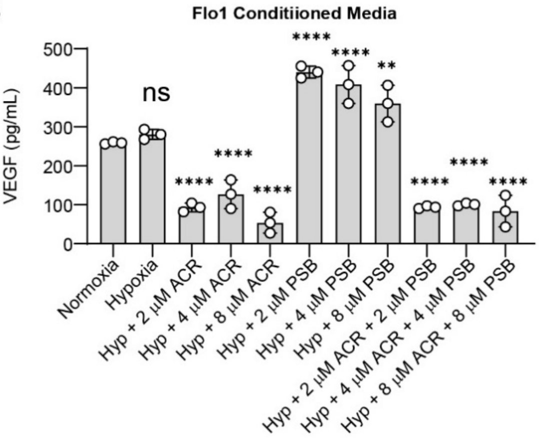

(A) HUVEC tube formation (in vitro angiogenesis) with conditioned media from treated FLO-1 cells treated with acriflavine and PSB12379. (B) VEGF levels in conditioned media after 48h treatment of FLO-1 cells with acriflavine, PSB12379 or their combination under hypoxic conditions.

WESTERN BLOT ORIGINAL IMAGES:

Figure 2D FLO-1

ponceau

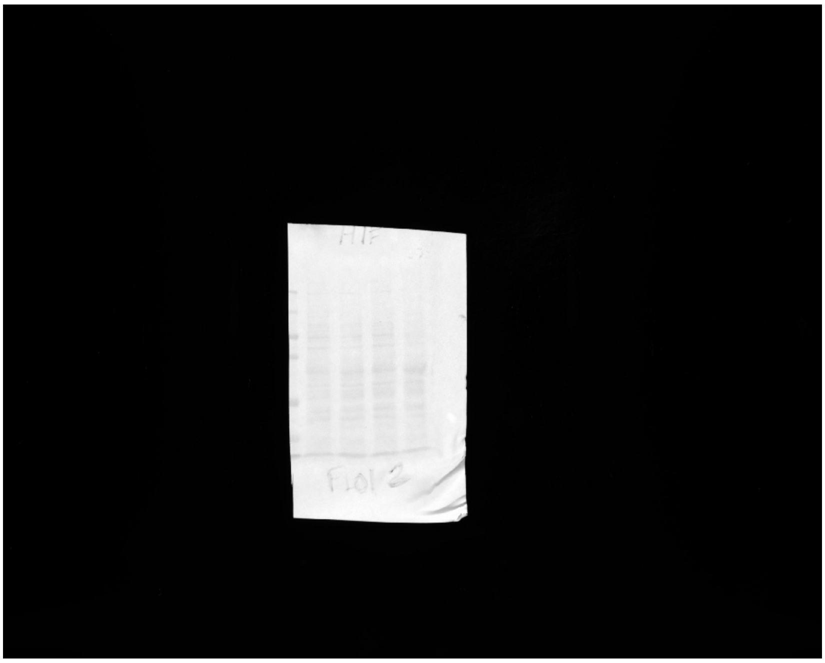

GAPDH

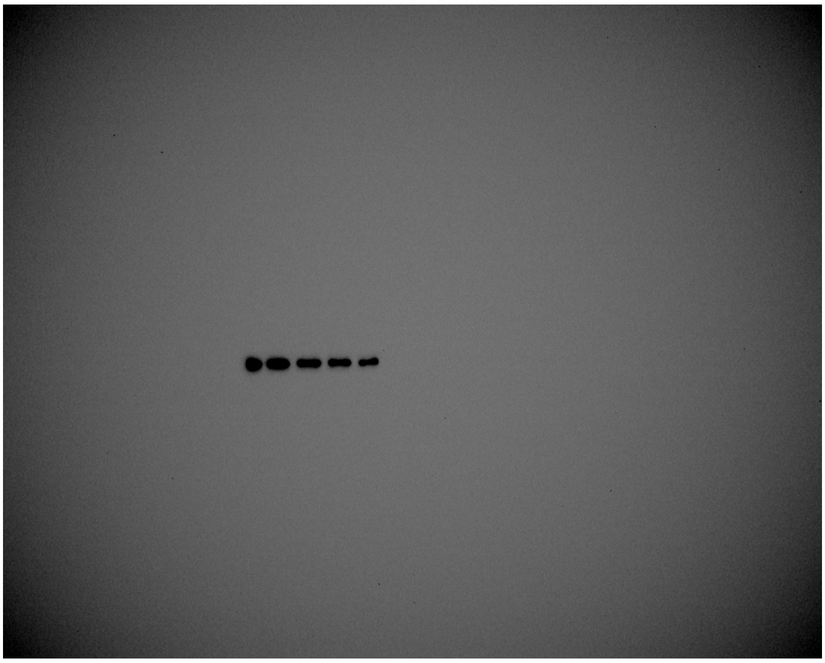

HIF1α

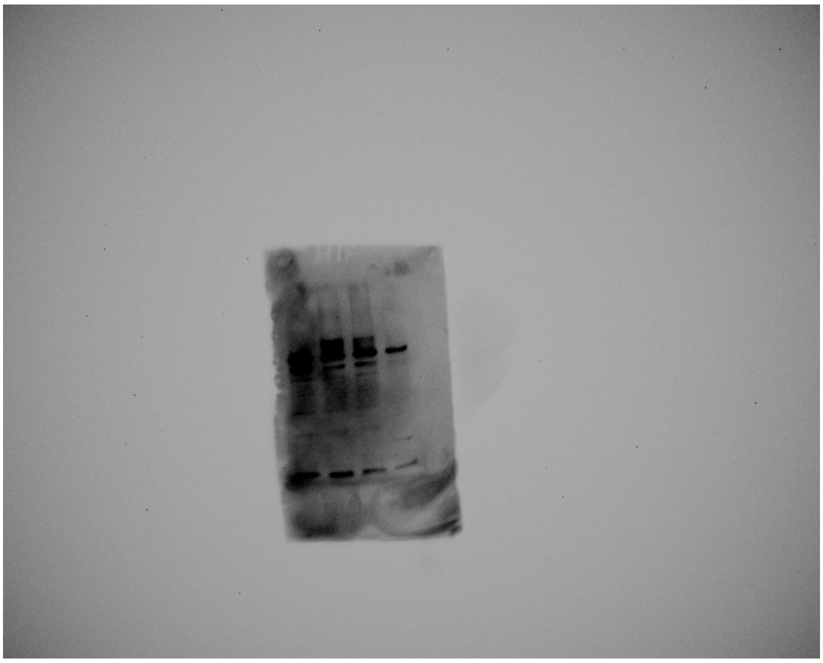

NT5E

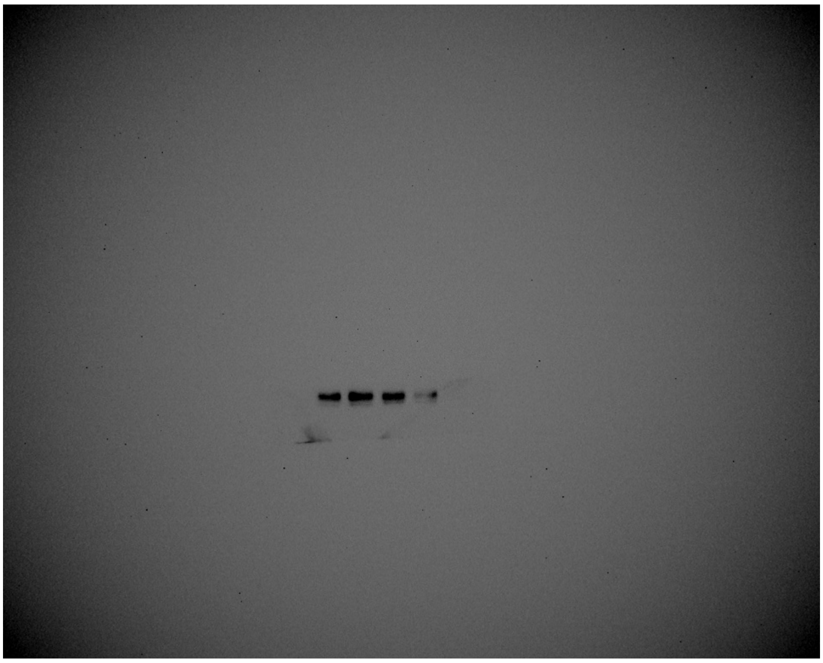

Supplementary Figure S2D OE19

Note: Lane 1 cropped out from figure panel, wrong sample added

GAPDH

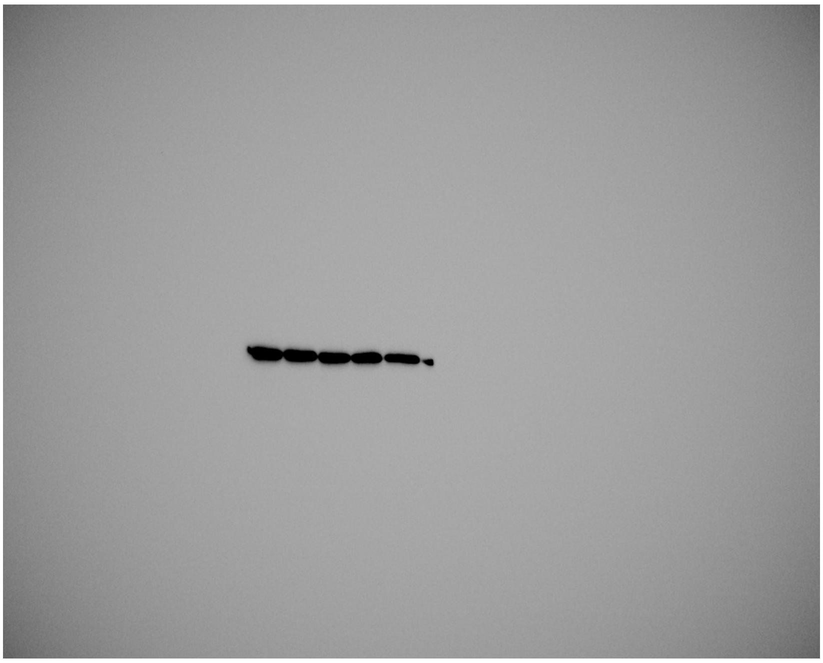

NT5E

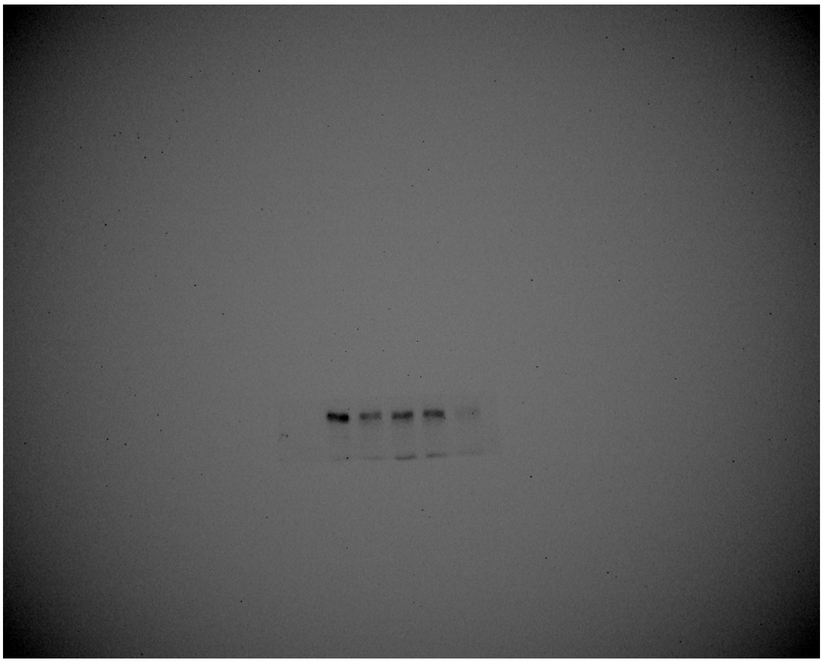

Supplementary Figure S2E OE33

GAPDH

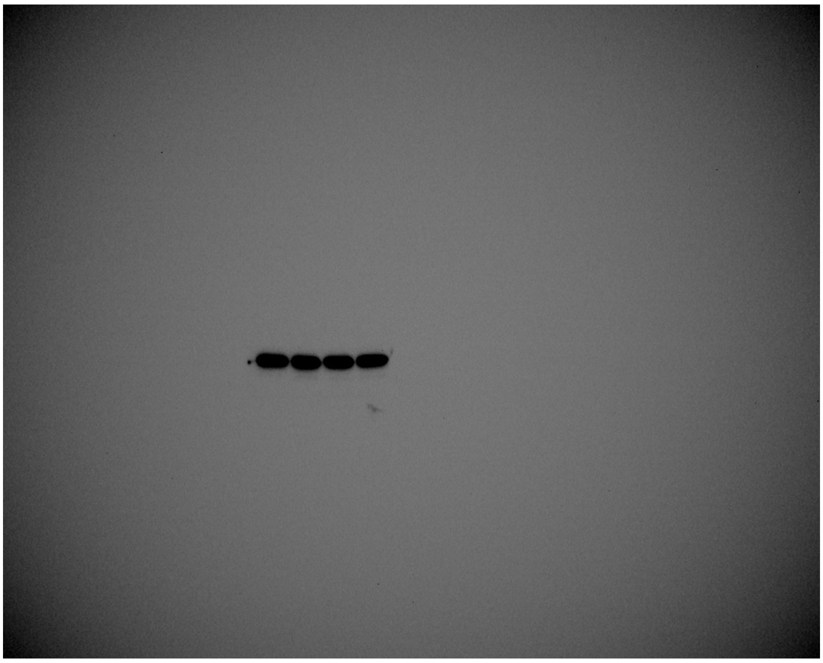

NT5E

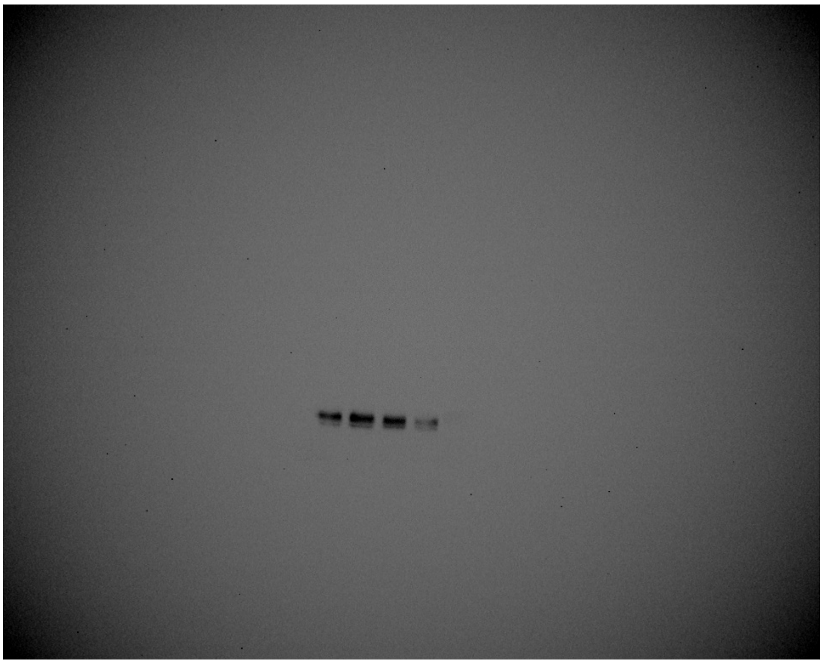

Additional HIF1α repeats

Top OE19

Middle OE33

Bottom FLO-1

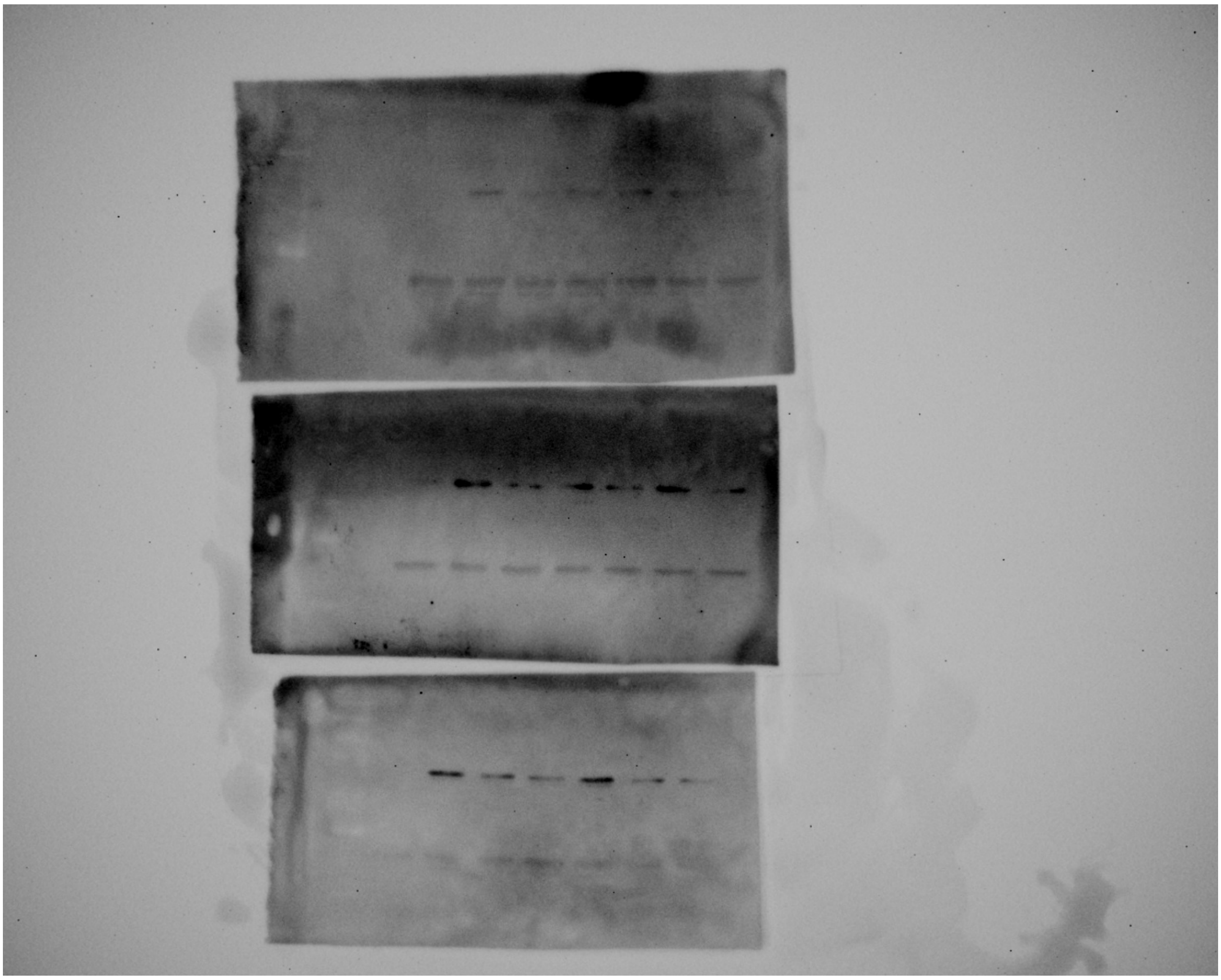

Lanes (left to right):

Lane 1 Control

Lane 2 Hypoxia (rep 2)

Lane 3 Hypoxia + 0.5 μM acriflavine (rep 2)

Lane 4 Hypoxia + 1 μM acriflavine (rep 2)

Lane 5 Hypoxia (rep 3)

Lane 6 Hypoxia + 0.5 μM acriflavine (rep 3)

Lane 7 Hypoxia + 1 μM acriflavine (rep 3)

FLO-1 Additional blot HIF1α (top band)

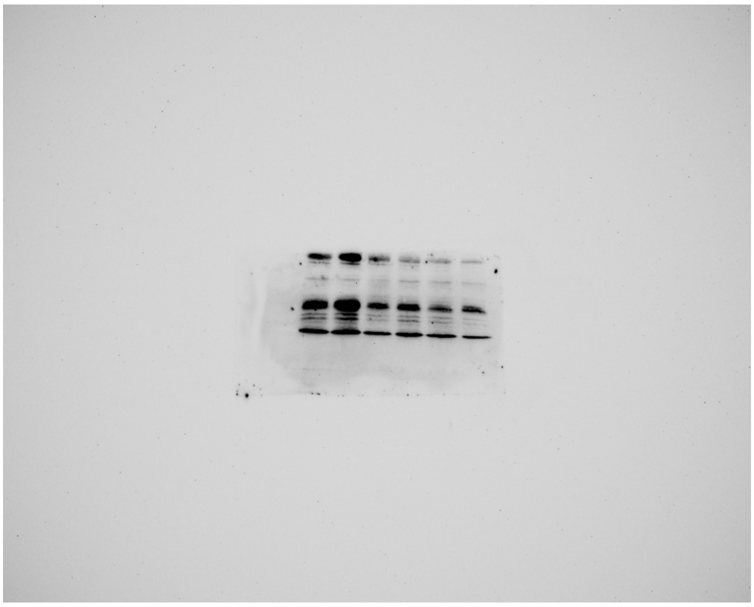

Lane 1 Control

Lane 2 Hypoxia

Lane 3 Hypoxia + 0.5 μM acriflavine

Lane 4 Hypoxia + 1 μM acriflavine

Lane 5 Hypoxia + 2 μM acriflavine

Lane 6 Hypoxia + 5 μM acriflavine

Western blot detailed images

FLO-1 Blots in Figure 2

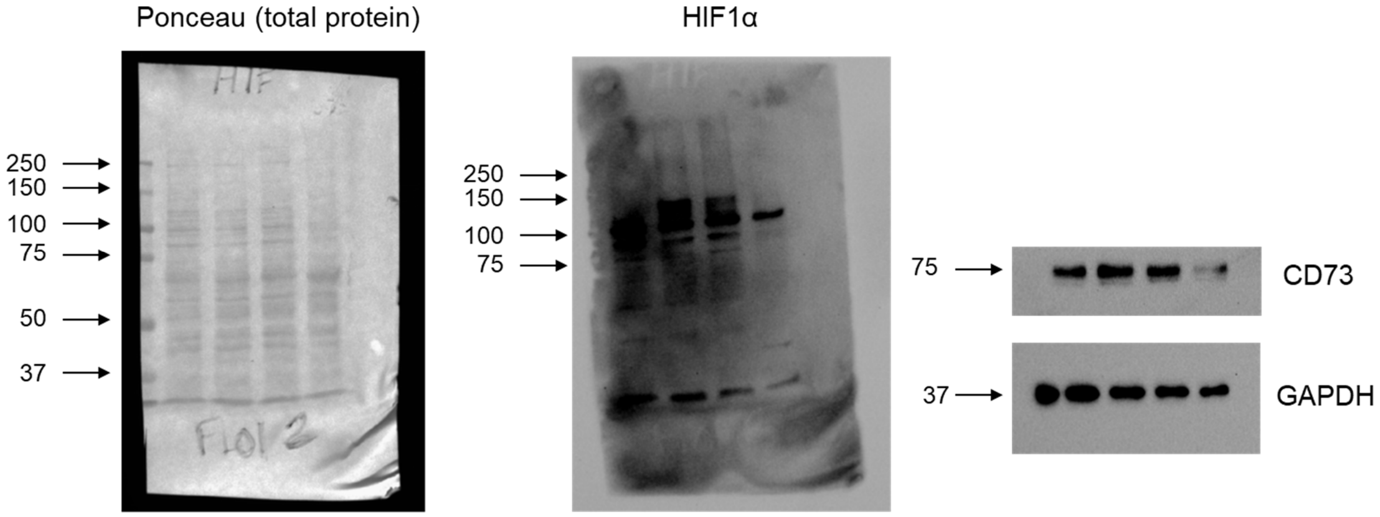

Note: ladder visible in GAPDH blot

Densitometry of above images

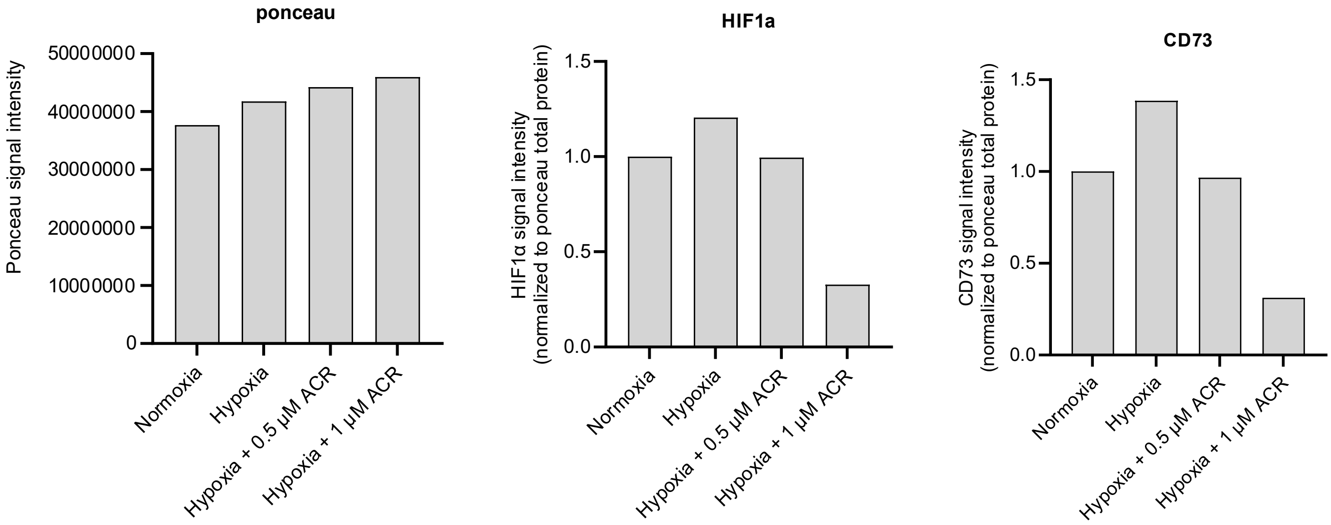

Note, GAPDH is often used as loading control for densitometry; however, it is regulated by HIF1a so ponceau total protein was used as loading control.

Additional HIF1α Blot for Figure 2 per Reviewer request

Ponceau (total protein)

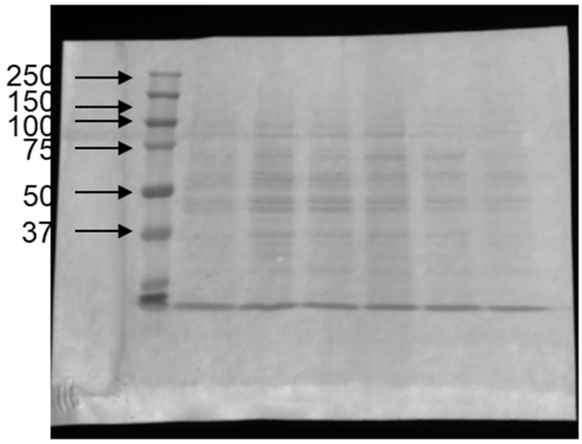

HIF1α and GAPDH

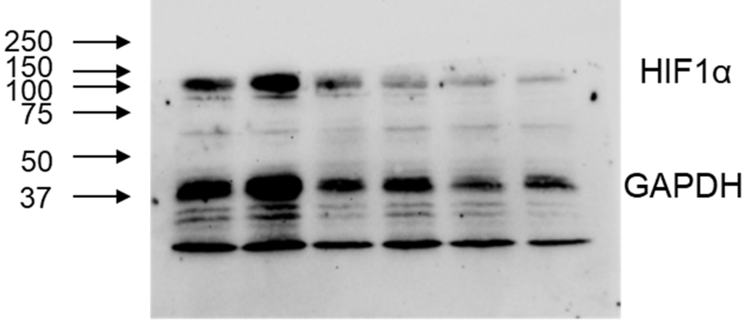

Lanes 5 and 6 were cropped out, they were higher concentrations of acriflavine that were too cytotoxic leading to loading control issues (see ponceau stain).

HIF1α replicate 2

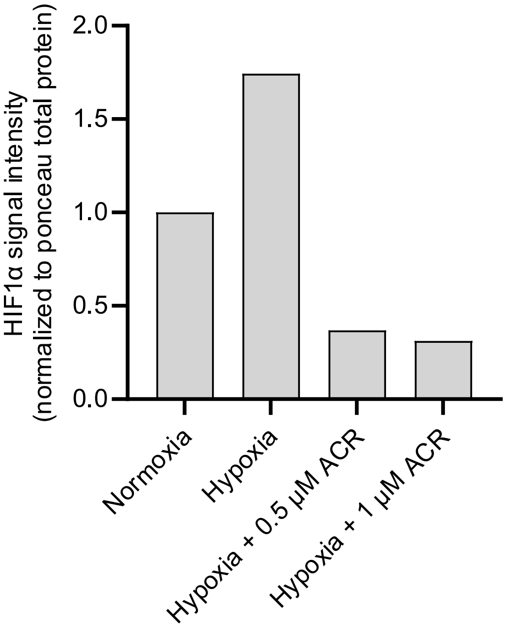

OE19 Blots in Supplementary Figure 2D

Lane 1 cropped out in Supplementary Figure 2D, unintended sample added to that lane.

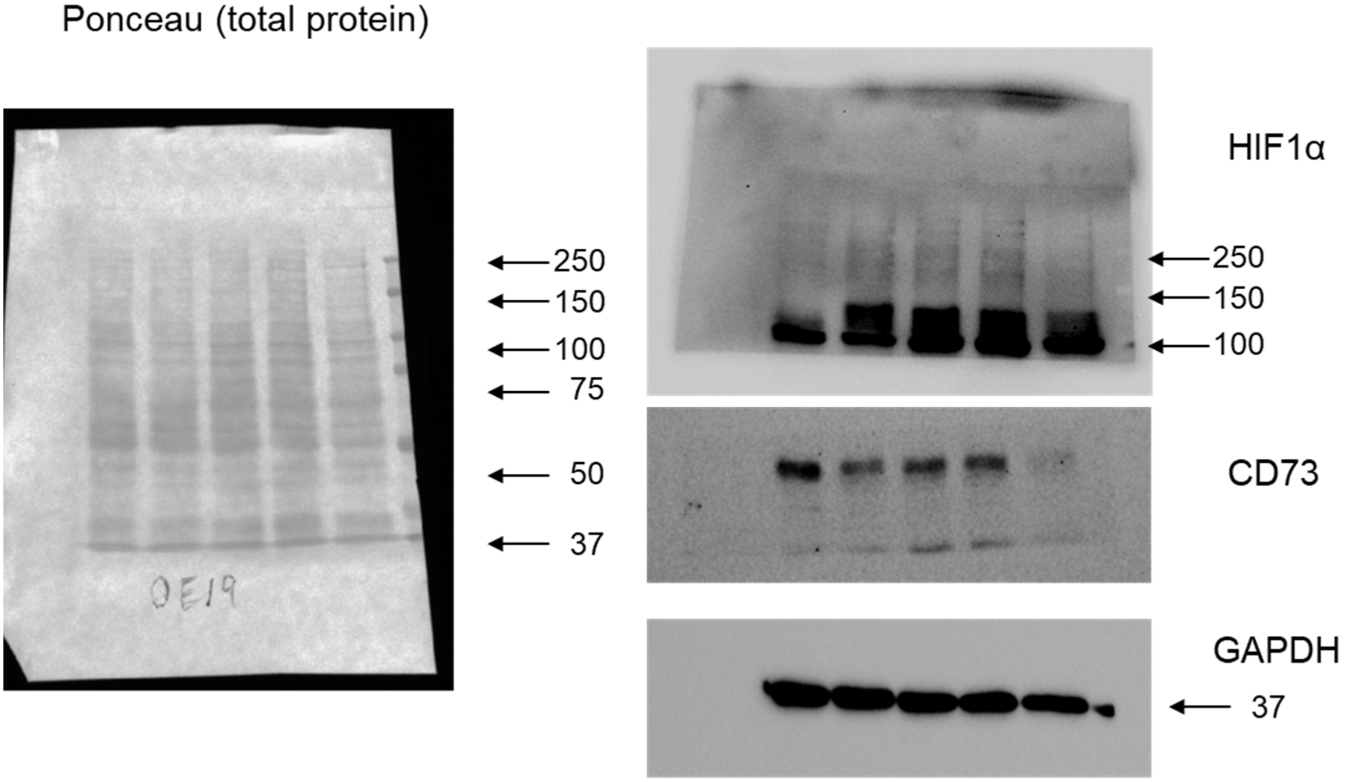

Note: ladder visible in GAPDH blot

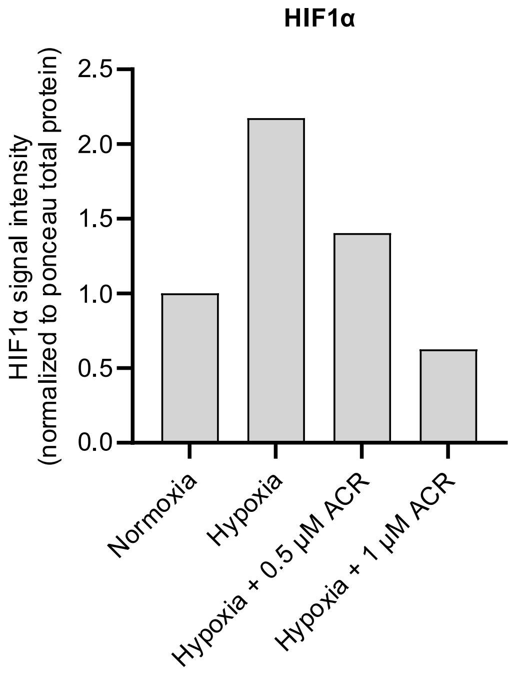

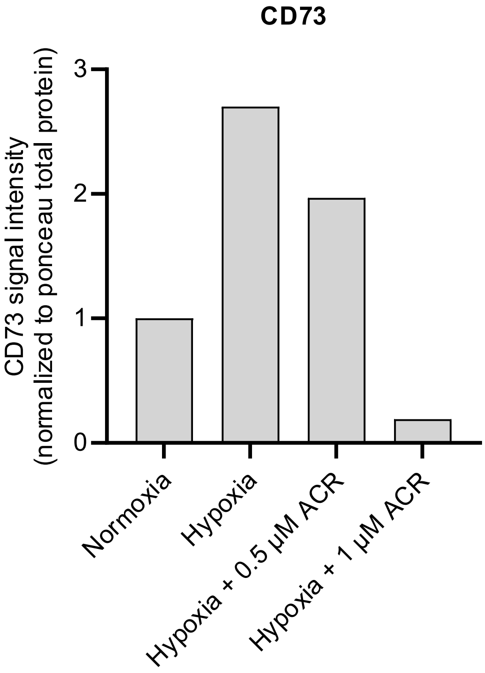

OE33 Blots in Supplementary Figure 2E and repeat

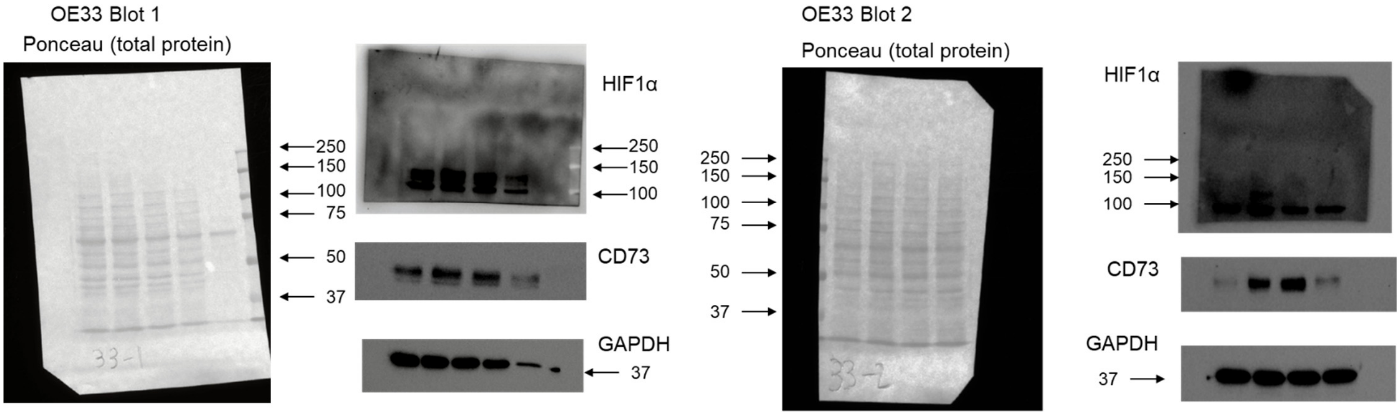

Example densitometry bands

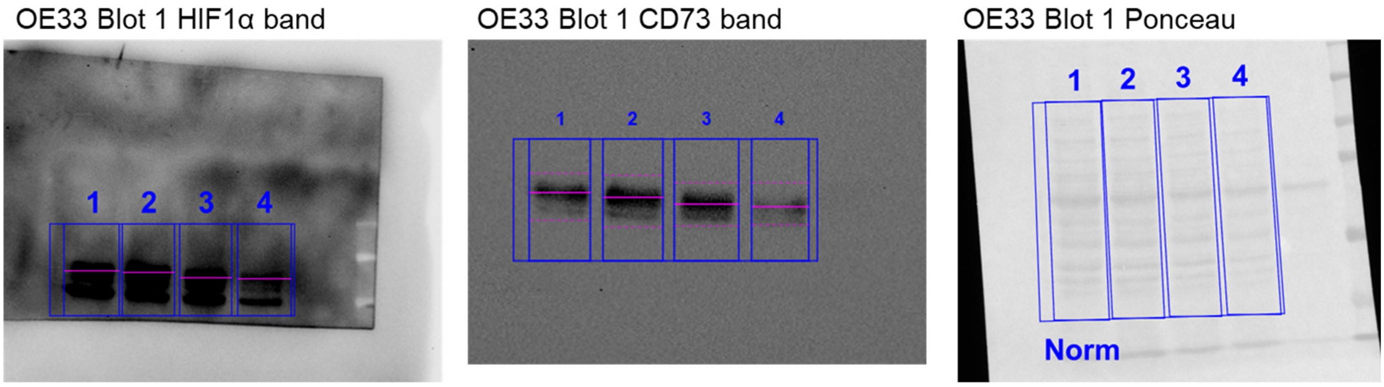

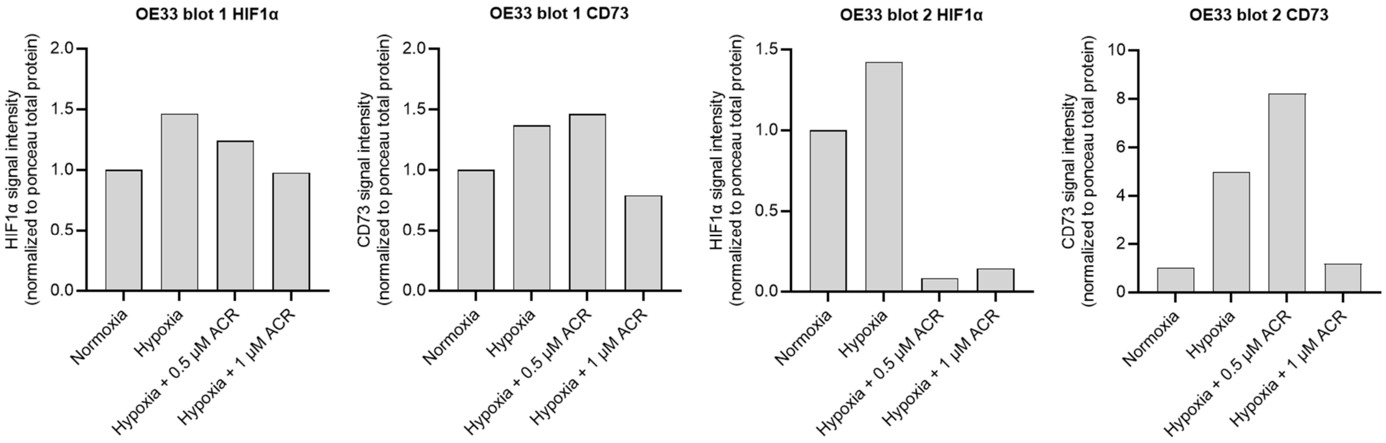

Supplement: Supplementary file 1 [file cancers-17-04016-s001.zip › cancers-3977129-supplementary.pdf]
